# Comparing dependent kappa coefficients obtained on multilevel data

**DOI:** 10.1002/bimj.201600093

**Published:** 2017-05-02

**Authors:** Sophie Vanbelle

**Affiliations:** ^1^ Methodology and Statistics CAPHRI, Maastricht University P. Debyeplein 1 6229 HA Maastricht The Netherlands

**Keywords:** Clustered bootstrap, Delta method, Hierarchical, Intraclass, Rater

## Abstract

Reliability and agreement are two notions of paramount importance in medical and behavioral sciences. They provide information about the quality of the measurements. When the scale is categorical, reliability and agreement can be quantified through different kappa coefficients. The present paper provides two simple alternatives to more advanced modeling techniques, which are not always adequate in case of a very limited number of subjects, when comparing several dependent kappa coefficients obtained on multilevel data. This situation frequently arises in medical sciences, where multilevel data are common. Dependent kappa coefficients can result from the assessment of the same individuals at various occasions or when each member of a group is compared to an expert, for example. The method is based on simple matrix calculations and is available in the R package “multiagree”. Moreover, the statistical properties of the proposed method are studied using simulations. Although this paper focuses on kappa coefficients, the method easily extends to other statistical measures.

## 1 Introduction

Reliability and agreement studies are of paramount importance in behavioral, social, biological, and health sciences. They both provide information about the quality of the measurements (Kottner et al., 2011). When observers classify items on a categorical scale, reliability refers to the ability of the scale to differentiate between the items, despite the presence of measurement error while agreement refers to the degree of closeness between two assessments made on the same items. Good reliability is an essential property of a measurement scale, especially when assessing the correlation with other variables because of the well‐known attenuation effect (that is, the presence of measurement error tends to weaken correlations between variables). In addition to good reliability, good agreement is sometimes also imperative, as in clinical decision making where observers should provide exactly the same scores, in order to make the same decision for the patient. Agreement is also involved in the assessment of criterion validity, where the degree of agreement between a measurement instrument under scrutiny and a reference method, which is often also subject to measurement error, is studied.

This paper is motivated by the third part of an exploratory study aimed at investigating the influence of different factors on inter‐ and intraobserver agreement levels on the evaluation of oropharyngeal dysphagia severity (Pilz *et al*., 2016). Oropharyngeal dysphagia is characterized by difficulties in swallowing. In addition to quality of life deterioration, it can have severe consequences such as malnutrition, dehydration, aspiration pneumonia, and sudden death. Fiberoptic endoscopic evaluation of swallowing (FEES) is nowadays the first choice method to evaluate the severity of oropharyngeal dysphagia. It permits the anatomical assessment of the pharyngeal and laryngeal structures and provides a comprehensive evaluation of the pharyngeal stage of swallowing. FEES consists of five criteria in the visual evaluation and interpretation of swallowing images: (1) valleculae pooling (No, <50%, ≥50%), (2) pyriform pooling (No, <50%, ≥50%), (3) number of piecemeal deglutitions (1, 2, 3, 4, or 5 or more often), (4) posterior spill (No, Yes) and (5) penetration/aspiration (No, <50%, ≥50%). Despite the increasing popularity of the FEES assessment, there is no standardization of the measurement criteria. Crucially, the interpretation of swallowing images is based on visual judgment and is thus subjective. It might be influenced by factors like observer's experience or bolus consistency. The FEES study therefore aimed to investigate the influence of different factors on inter‐ and intraobserver agreement levels.

In the third FEES study part, two observers (medical students who received a special training) independently assessed 40 swallowing images obtained on 20 patients, who consecutively swallowed 5cc of a thin liquid and 5cc of a thick liquid. The swallowing images were assessed in a random order by the observers, blinded to any medical information on the patient. The exercise was repeated after two weeks under the same conditions to determine the intraobserver agreement level of each observer. Then, the two observers reviewed the medical images during two consensus meetings, planned two weeks apart. During the consensus meetings, the two students reviewed the images together and determined a score in consensus. The aim was to compare the individual intraobserver agreement level of the two students to the intraobserver agreement level obtained by the students in consensus.

The FEES study possesses two particularities. First, the structure of the data is multilevel, that is items are nested within clusters. Here, two swallows (one with thin and one with thick liquid) are nested within patients. Multilevel data are common in medical and behavioral sciences, where measures are often obtained on persons nested in organizations (e.g. patients in health care centers), on different body parts or by repeated measurements over time. Ignoring the multilevel structure of the data can lead to incorrect conclusions (see e.g. Hox, 2002). Secondly, the same patients were evaluated by the same observers under two experimental conditions (individually and in consensus). This then introduces a dependency between the agreement coefficients to be compared, a dependency that also needs to be taken into account.

When items (subjects/objects) are evaluated by observers on a categorical scale, then reliability, as classically defined, can be measured through the intraclass kappa coefficient for binary scales (Kraemer, 1979). For ordinal scales, Cohen (1968), Fleiss and Cohen (1973), and Schuster (2004) showed that the quadratic weighted kappa coefficient is asymptotically equivalent to an intraclass correlation coefficient. However, for nominal scales, reliability has to be assessed separately for each category with the intraclass kappa coefficient (Kraemer, 1979). On another hand, for nominal scales, agreement can be measured through Cohen's kappa coefficient (Cohen, 1960) and for ordinal scales, through the linear weighted kappa coefficient (Cohen, 1968; Cicchetti and Allison, 1971; Vanbelle, 2016). Kappa coefficients are relative agreement coefficients. They have the particularity to involve the marginal probability distribution of the observers, that is the probability for an observer to classify items in the different categories of the scale (Warrens, 2010, 2014). Through this relationship, kappa coefficients depend on the prevalence of the trait under study, which limits the possibility to compare them among studies with different prevalence. Several authors (Thompson and Walter, 1988; Feinstein and Cicchetti, 1990; Cicchetti and Feinstein, 1990; Byrt *et al*., 1993; de Vet *et al*., 2006) proposed the use of absolute agreement measures (e.g. the proportion of items classified in the same category by the two observers) to avoid that dependency. These absolute coefficients are however not sensitive to the scales' inability in distinguishing between items in a population with low prevalence and kappa coefficients are therefore to be preferred (Rogot and Goldberg, 1966; Vach, 2005; Kraemer *et al*., 2004; Vanbelle, 2016).

While the statistical analysis of multilevel data became very popular in the last decades, only little attention was paid to the evaluation of agreement in the presence of multilevel data. This could be explained by the fact that it is common practice to summarize the information at the highest level of the hierarchy (e.g. the patient in the FEES study) following rules established by the researchers and then compute agreement based on the summary measures. For example, the FEES score could be defined at patient level as the average or the maximum score obtained for the thin and the thick swallow. By doing so, information is lost on possible disagreements at the lowest level of the hierarchy and this can result in biased estimates of agreement levels. Moreover, it is not possible to predict the relationship between the agreement values obtained at different hierarchical levels (Vanbelle *et al*., 2012).

Kappa coefficients were nevertheless extended over the years to account for particular study designs. In particular, population‐averaged (Thomson, 2001; Williamson and Manatunga, 1997; Williamson *et al*., 2000; Gonin *et al*., 2000) and unit‐specific models (Gajewski *et al*., 2007; Vanbelle *et al*., 2012; Vanbelle and Lesaffre, 2016) were developed to account for a multilevel data structure and for the presence of categorical and continuous predictors. While these modeling techniques represent a considerable progress, they require adequate model specifications, expert programming skills, and a reasonable sample size (Carey *et al*., 1993). The latter is not achieved with the 20 patients of the FEES study.

Recently, Yang and Zhou (2014, 2015) developed a marginal approach, based on the delta method, involving only simple matrix calculations to adjust the standard error of kappa coefficients in the presence of multilevel data. Their derivations are however limited to the estimation of a single kappa coefficient and make the comparison of several dependent kappa coefficients impossible. Dependent kappa coefficients can occur in many ways. For example, two observers may assess the same individuals at various occasions or in different experimental conditions like in the FEES study. Alternatively, each member of a group of observers may be compared to an expert in assessing the same items on a categorical scale. In this latter case, the agreement coefficient is used as criterion validity measure. In the present paper, we therefore develop a method to compare several dependent kappa coefficients obtained on multilevel data. This provides a new practical and simple alternative to the more advanced statistical techniques. The alternative method is based on the use of Hotelling's *T* ^2^ statistic, previously used to compared dependent kappa coefficients (Vanbelle and Albert, 2008). This paper improves the earlier method extending to multilevel data structures and using two different ways to estimate the variance‐covariance matrix between the kappa coefficients. The variance‐covariance matrix is derived using the delta method and the clustered bootstrap method (Field and Welsh, 2007).

The kappa coefficients are introduced in Section 2. In Section 3, the kappa coefficients are generalized to multilevel structures (Yang and Zhou, 2014, 2015). Further, the method to compare several kappa coefficients is provided in Section 4 using the delta method and the clustered bootstrap method. The statistical properties of the new method are studied in Section 5 for a binary and a 3‐ordinal scale. In Section 6, the otorhinolaryngological data are analyzed. Finally, the method is discussed in Section 7.

## 2 Definition of the kappa coefficients

Kappa coefficients were initially defined in terms of computation procedure rather than population parameters (see e.g. Kraemer, 1979). Vanbelle (2016) provided recently a definition in terms of population parameters making the interpretation of the most common kappa forms straightforward. This definition will be adopted here.

Consider a population of items (subjects or objects) I and two fixed observers. In the FEES study, the items are swallowing images and the two observers are medical students. Let the random variable $Y_{kr}$ represent the classification of item *k* by observer *r*, that is $Y_{kr}=i$ if observer *r* (r=1,2) classifies a randomly selected item *k* of population I in category *i* (i=1,⋯,g). Further consider the random variable $Z_{k}=f\left(Y_{k1},Y_{k2}\right)$ representing the disagreement between the two observers on the classification of item *k*. When the scale is binary or nominal, the function $f\left(Y_{k1},Y_{k2}\right)=1-I\left(Y_{k1},Y_{k2}\right)$ is usually used, where *I*(., .) is the identity function. The random variable $Z_{k}$ then equals 1 if a disagreement occurs and equals 0 otherwise. When the scale is ordinal, functions of the form $f\left(Y_{k1},Y_{k2}\right)={\left|Y_{k1}-Y_{k2}\right|}^{s}$ (s∈N) are usually used. In practice, s=1 or s=2 is most common. The random variable $Z_{k}$ then gives the distance (number of categories) separating the classifications made by the two observers when s=1. This number is squared when s=2.

Kappa coefficients are defined by the formula

(1) κ=1−E(Zk)E ind (Zk),

where $E(Z_{k})$ is the expectation of $Z_{k}$ over the population of items and E ind (Zk) is the expectation assuming statistical independence of the ratings made by the two observers, that is $P\left(Y_{k1}=i,Y_{k2}=j\right)=P\left(Y_{k1}=i\right)P\left(Y_{k2}=j\right)$.

When the function $f\left(Y_{k1},Y_{k2}\right)=1-I\left(Y_{k1},Y_{k2}\right)$ is used, Cohen's kappa coefficient is obtained. Cohen's kappa coefficient compares the expected probability of disagreement to the same probability under the statistical independence of the ratings. Using $f\left(Y_{k1},Y_{k2}\right)={\left|Y_{k1}-Y_{k2}\right|}^{s}$ leads to the linear weighted kappa coefficient when s=1 and to the quadratic kappa coefficient when s=2. The linear (quadratic) weighted kappa coefficient compares the expected (squared) number of categories separating the classifications made by the two observers to the same number under the statistical independence of the ratings. Kappa coefficients are therefore relative agreement measures, depending on the marginal probability distribution of the observers $P(Y_{k1}=i)$ and $P(Y_{k2}=j)$ (∀i,j∈1,⋯,g) through the denominator of Eq. (1). Kappa coefficients vary between −1 and 1. The value 1 is reached when there is perfect agreement between the two observers while a value of 0 means that the agreement is equal to what is expected under the statistical independence of the ratings.

The quantity $E(Z_{k})$ can be expressed according to the joint classification probabilities of the two observers $P(Y_{k1}=i,Y_{k2}=j)$ using agreement weights $w_{ij}$ or disagreement weights $v_{ij}$. Suppose that the joint probabilities are the same for all items in the population, that is $P\left(Y_{k1}=i,Y_{k2}=j\right)=\pi_{ij}$. This implies that the marginal probability distribution for observer 1 is given by $\pi_{i+}=\sum_{j=1}^{g}\pi_{ij}$ and for observer 2 by $\pi_{+j}=\sum_{i=1}^{g}\pi_{ij}$. Then, the kappa coefficient can be expressed as

(2) $$\kappa =1-\frac{Q_{o}}{Q_{e}}=\frac{\Pi_{o}-\Pi_{e}}{1-\Pi_{e}},$$

with $Q_{o}=\sum_{i=1}^{g}\sum_{j=1}^{g}v_{ij}\pi_{ij}$ and $\Pi_{o}=\sum_{i=1}^{g}\sum_{j=1}^{g}w_{ij}\pi_{ij}$. The quantities $Q_{e}$ and $\Pi_{e}$ are obtained by replacing $\pi_{ij}$ by $\pi_{i+}\pi_{+j}$ in $Q_{o}$ and $\Pi_{o}$, respectively.

The agreement weights $w_{ij}=I\left(i,j\right)$ were introduced by Cohen (1960). Cicchetti and Allison (1971) introduced the linear agreement weights $w_{ij}=1-\left|i-j\right|/\left(g-1\right)$ and Cohen (1968) the quadratic agreement weights $w_{ij}=1-{\left[\left(i-j\right)/\left(g-1\right)\right]}^{2}$. For binary scales, under the assumption of equal marginal probability distributions ($\pi_{i+}=\pi_{+i}$ ∀i∈1,⋯,g), Cohen's kappa coefficient is called the intraclass kappa coefficient and is a reliability measure (Kraemer, 1979). That is, the intraclass kappa coefficient is the ratio of the variance of the “true” scores to that of the observed scores where the “true” score is the mean over independent replications of the measure (Kraemer *et al*., 2004). The quadratic kappa coefficient was also shown to be asymptotically equivalent to an intraclass correlation coefficient (Cohen, 1968; Fleiss and Cohen, 1973; Schuster, 2004).

## 3 Definition of multilevel kappa coefficients

Suppose now that the population I possesses a 2‐level hierarchical structure in the sense that observations are made on $n_{k}$ items (level 1 of the hierarchy) nested in *K* clusters (level 2 of the hierarchy) ($\sum_{k=1}^{K}n_{k}=N$). In the FEES example, the clusters are the patients and there are two swallows with a different liquid consistency nested in each patient.

In order to define an overall kappa coefficient over the population of items, Yang and Zhou (2014) make two assumptions. First, they assume that the members of a cluster are homogeneous, in the sense that each member of cluster *k* has the same probability $\pi_{ij,k}$ of being classified in category *i* by observer 1 and *j* by observer 2. The identity shows that the members have the same probability to be classified in category *i* by rater 1 ($\pi_{i+,k}$) and in category *j* by rater 2 ($\pi_{+j,k}$), with $\pi_{i+,k}=\sum_{j=1}^{g}\pi_{ij,k}$ and $\pi_{+j,k}=\sum_{i=1}^{g}\pi_{ij,k}$. In the FEES study, this means that the oropharyngeal dysphagia severity scores should not depend on the liquid consistency. Secondly, Yang and Zhou (2014) assume the homogeneity of the pairwise classification among the *K* clusters, that is $E\left(\pi_{ij,k}\right)=\pi_{ij}$ and therefore of the marginal classification probabilities ($E\left(\pi_{i+,k}\right)=\pi_{i+}$ and $E\left(\pi_{+j,k}\right)=\pi_{+j}$). In the FEES study, this means that all patients should possess the same probability to be classified in the different severity categories, that is that there is no patient sub‐population in terms of dysphagia severity.

Let $\nu_{k}=n_{k}/N$ denote the relative sample size of the *k*‐th cluster. In the FEES study, $\nu_{k}=2/N$ since there are two swallows per patient. The marginal probability distribution of an observer is the weighted average of the marginal probability distribution at the cluster level, that is $\pi_{i+}=\sum_{k=1}^{K}\nu_{k}\pi_{i+,k}$ and $\pi_{+j}=\sum_{k=1}^{K}\nu_{k}\pi_{+j,k}$. In the same way, the pairwise classification probabilities over the population of clusters are given by $\pi_{ij}=\sum_{k=1}^{K}\nu_{k}\pi_{ij,k}$. The weighted kappa coefficient for multilevel data is then defined as (Yang and Zhou, 2014)

$$\kappa =\frac{\Pi_{o}-\Pi_{e}}{1-\Pi_{e}}$$

where $\Pi_{o}=\sum_{i=1}^{g}\sum_{j=1}^{g}w_{ij}\pi_{ij}$ is the agreement and $\Pi_{e}=\sum_{i=1}^{g}\sum_{j=1}^{g}w_{ij}\pi_{i+}\pi_{+j}$ the agreement expected under the statistical independence assumption of the ratings, that is $\pi_{ij}=\pi_{i+}\pi_{+j}$. Note that $\Pi_{o}$ can be rewritten as

$$\Pi_{o}=\sum_{i=1}^{g}\sum_{j=1}^{g}w_{ij}\sum_{k=1}^{K}\nu_{k}\pi_{ij,k}=\sum_{k=1}^{K}\nu_{k}\sum_{i=1}^{g}\sum_{j=1}^{g}w_{ij}\pi_{ij,k}=\sum_{k=1}^{K}\nu_{k}\Pi_{o,k}.$$

This implies that the agreement is a weighted average of the agreement obtained at the cluster level.

The weights $w_{ij}$ presented in the above equation are the same as those defined in Section 2, to lead to the multilevel counterparts of Cohen's, the linear and the quadratic kappa coefficients. Yang and Zhou (2014) showed that the weighted kappa coefficient obtained with the quadratic weights can be interpreted as an intraclass correlation coefficient and is a reliability measure. Using the linear weights, the weighted kappa coefficient is a relative agreement measure comparing the mean distance between the classification of the two observers to the mean distance under the independence assumption of the ratings (Vanbelle, 2016). The family of kappa coefficients as defined by Yang and Zhou (2014) corresponds to the classical kappa coefficients when the hierarchical level of the data is ignored. A sample estimate of the kappa coefficients is obtained by replacing the probabilities $\pi_{ij,k}$, $\pi_{i+,k,}$ and $\pi_{+j,k}$ by their corresponding sample proportions $p_{ij,k}$, $p_{i+,k}$ and $p_{+j,k}$.

## 4 Comparison of several dependent kappa coefficients

### 4.1 Hotelling's *T* ^2^ test

Hotelling's *T* ^2^ test will be used (Vanbelle and Albert, 2008) to compare several dependent multilevel coefficients defined in Section 3. Suppose that L≥2 dependent kappa coefficients ($\kappa_{1},\cdots ,\kappa_{L}$) obtained on multilevel data have to be compared. That is, we wish to test the null hypothesis $H_{0}:C\kappa =0$ versus $H_{1}:C\kappa \neq 0$, where $\kappa ={\left(\kappa_{1},\cdots ,\kappa_{L}\right)}^{T}$ and *C* is a (L−1)×L patterned matrix obtained by merging the (L−1)×(L−1) identity matrix and a (L−1)×1 vector of −1. For example, in the FEES study, we are interested in comparing three kappa coefficients. One kappa coefficient is obtained between the measurements made two weeks apart for each medical student individually (namely, κ_1_ and κ_2_) and one kappa coefficient is obtained between the two consensus meetings of the two students (namely, κ_3_). This yields to

$$\left(\begin{array}{c} \kappa_{1}-\kappa_{3}\\ \kappa_{2}-\kappa_{3} \end{array}\right)=\left(\begin{array}{ccc} 1 & \;0 & \;-1\\ 0 & \;1 & \;-1 \end{array}\right)\left(\begin{array}{c} \kappa_{1}\\ \kappa_{2}\\ \kappa_{3} \end{array}\right)=C\kappa .$$

The test statistic

(3) $$T^{2}={\left(C\;\hat{\kappa }\right)}^{T}{\left(CSC^{T}\right)}^{-1}C\;\hat{\kappa },$$

where $\hat{\kappa }$ and *S* are respectively a vector of estimates of ***κ*** and their estimated variance‐covariance matrix, is distributed as Hotelling's *T* ^2^ under two assumptions. The first is the existence of a common kappa coefficient across the clusters. This assumption is already made by Yang and Zhou (2014). The second assumption is multivariate normality of the vector of kappa coefficients ***κ***. The null hypothesis is rejected at the α‐level if

(4) $$T^{2}\geq \frac{\left(K-1)(L-1\right)}{\left(K-L+1\right)}Q_{F}\left(1-\alpha ;L-1,K-L+1\right)$$

where $Q_{F}\left(1-\alpha ;L-1,K-L+1\right)$ is the upper α‐percentile of the F distribution on L−1 and K−L+1 degrees of freedom. Note that, since “K−L+1” is large in general, the left‐hand side of Eq. 4 can be approximated by $Q_{\chi^{2}}\left(1-\alpha ;L-1\right)$, the (1−α)‐th percentile of the chi‐square distribution on L−1 degrees of freedom. If $c_{l}$ denotes the *l*‐th row of matrix *C*, multiple comparisons can be made by using simultaneous confidence intervals for contrasts $c_{l}^{T}\kappa$ (l=1,⋯,L−1), namely

$$c_{l}^{T}\hat{\kappa }\pm \sqrt{\frac{\left(K-1)(L-1\right)}{\left(K-L+1\right)}Q_{F}\left(1-\alpha ;L-1,K-L+1\right)}\sqrt{c_{l}^{T}Sc_{l}}.$$

Note that other forms of the matrix *C* can be envisaged, depending on the individual contrasts of interest.

### 4.2 The delta method

Yang and Zhou (2014, 2015) determined the asymptotic variance of a single multilevel kappa coefficient with the delta method. In this section, we will apply the delta method twice to derive the asymptotic variance‐covariance matrix *S*, involved in Eq. 3. For notation convenience, the asymptotic variance‐covariance matrix will be derived for the comparison of L=2 kappa coefficients. The extension to more than two kappa coefficients is straightforward since the covariance is defined on pairs of variables.

**Asymptotic variance‐covariance of the observed and expected agreements** Let $p_{rstu,k}$ be the proportion of items from cluster *k* classified in category *r* by observer 1, *s* by observer 2, *t* by observer 3 and *u* by observer 4 and suppose that we are interested in the comparison of the agreement coefficient obtained between observers 1 and 2 to the agreement coefficient obtained between observers 3 and 4. Let $p_{•+++,k}$, $p_{+•++,k}$, $p_{++•+,k}$ and $p_{+++•,k}$ be the vectors with the marginal classification proportions relative to cluster *k* for the observers 1, 2, 3, and 4, respectively. For example, $p_{•+++,k}={\left(p_{1+++,k},\cdots ,p_{g+++,k}\right)}^{T}$. Let $p_{rs++,k}$ and $p_{++tu,k}$ denote the proportions in the joint classification table relative to observers 1 and 2 and to observers 3 and 4, respectively. The observed agreement between observers 1 and 2 and observers 3 and 4 are respectively estimated by

$$P_{o1,k}=\sum_{r=1}^{g}\sum_{s=1}^{g}w_{rs}p_{rs++,k}\;\;\text{and}\;\;P_{o2,k}=\sum_{t=1}^{g}\sum_{u=1}^{g}w_{tu}p_{++tu,k}.$$

where $w_{ij}$ are agreement weights (i,j=1,⋯,g). Define the vector $\hat{\xi }$ as

$$\hat{\xi }=\left(\begin{array}{c} P_{o1}\\ P_{o2}\\ p_{•+++}\\ p_{+•++}\\ p_{++•+}\\ p_{+++•} \end{array}\right)=\sum_{k=1}^{K}\nu_{k}\left(\begin{array}{c} P_{o1,k}\\ P_{o2,k}\\ p_{•+++,k}\\ p_{+•++,k}\\ p_{++•+,k}\\ p_{+++•,k} \end{array}\right).$$

Similarly to Yang and Zhou (2014), it can be shown that asymptotically, under mild regular conditions, $\hat{\xi }$ is asymptotically normally distributed with variance‐covariance matrix var$\left(\hat{\xi }\right)$. The elements of var$\left(\hat{\xi }\right)$ are estimated in Appendix 1, following the technique of Obuchowski (1998).

To determine the two kappa coefficients to be compared, the expected agreement is also required for the two pairs of observers (namely, $P_{e1}$ and $P_{e2}$). In matrix notation, the expected agreement between observers 1 and 2 and between observers 3 and 4 is given by

$$P_{e1}=p_{•+++}^{T}\Lambda p_{+•++}\;\;\text{and}\;\;P_{e2}=p_{++•+}^{T}\Lambda p_{+++•}$$

where Λ is the g×g matrix with the agreement weights $w_{ij}$ as elements. The vector $\hat{\Psi }={\left(P_{o1},P_{o2},P_{e1},P_{e2}\right)}^{T}$ is a function of the vector $\hat{\xi }$ (i.e. $\hat{\Psi }=f\left(\hat{\xi }\right)$) fulfilling the conditions of the multivariate delta method. The asymptotic variance‐covariance matrix of $\sqrt{N}{\left(P_{o1},P_{o2},P_{e1},P_{e2}\right)}^{T}$ is, by application of the multivariate delta method, given by

$$\text{var}\left(\hat{\Psi }\right)=KJ\text{var}\left(\hat{\xi }\right)J^{T}$$

where *J* is the Jacobian matrix corresponding to *f*(.) with respect to $\hat{\xi }$, that is,

$$J=\left(\begin{array}{cccccc} 1 & \;0 & \;0^{T} & \;0^{T} & \;0^{T} & \;0^{T}\\ 0 & \;1 & \;0^{T} & \;0^{T} & \;0^{T} & \;0^{T}\\ 0 & \;0 & \;p_{+•++}^{T}\Lambda^{T} & \;p_{•+++}^{T}\Lambda & \;0^{T} & \;0^{T}\\ 0 & \;0 & \;0^{T} & \;0^{T} & \;p_{+++•}^{T}\Lambda^{T} & \;p_{++•+}^{T}\Lambda \end{array}\right)$$

and the vector ***0*** is the g×1 vector of zeros.

**Asymptotic variance‐covariance of the kappa coefficients**. In the same way, the vector of kappa coefficients $\hat{\kappa }={\left({\hat{\kappa }}_{1},{\hat{\kappa }}_{2}\right)}^{T}$ is a function of the vector $\hat{\Psi }$ fulfilling the conditions of the multivariate delta method, $\hat{\kappa }=h\left(\hat{\Psi }\right)$. The variance‐covariance matrix of $\hat{\kappa }$ is, by application of the multivariate delta method, given by

(5) $$\text{var}\left(\hat{\kappa }\right)=\hat{S}=\frac{1}{K}V\text{var}\left(\hat{\Psi }\right)V^{T}$$

with

$$V=\left(\begin{array}{cccc} \frac{1}{1-P_{e1}} & \;0 & \;\frac{P_{o1}-1}{{\left(1-P_{e1}\right)}^{2}} & \;0\\ 0 & \;\frac{1}{1-P_{e2}} & \;0 & \;\frac{P_{o2}-1}{{\left(1-P_{e2}\right)}^{2}} \end{array}\right).$$

The elements of $\text{var}(\hat{\kappa })$ are also given in Appendix 1. When there is only one unit per cluster ($n_{k}=1$ ∀k), the variance‐covariance matrix given by Eqn. 5 reduces to the classical variance‐covariance matrix multiplied by a correction factor, namely K/(K−1).

### 4.3 The clustered bootstrap method

Kang *et al*. (2013) determined the asymptotic variance of a single Cohen's kappa coefficient using clustered bootstrap. We will use this technique to determine the asymptotic variance‐covariance matrix *S* of *L* multilevel kappa coefficients, defined in Eq. 3. The clustered bootstrap consists of three steps:

1. Draw a random sample with replacement of size *K* from the cluster indexes.
2. For each cluster, select all observations belonging to the cluster. If the cluster sizes are different, the sample size of the bootstrap sample could be different from the original sample size *N*.
3. Repeat steps 1 and 2 to generate a total of *B* independent bootstrap samples.

For each bootstrap sample (b=1,⋯,B), the *L* multilevel kappa coefficients to be compared are determined, $\kappa_{1}^{b},\cdots ,\kappa_{L}^{b}$. The bootstrap estimate of the vector of kappa coefficients is then defined by Kang *et al*. (2013) as

(6) $${\hat{\kappa }}_{B}={\left({\hat{\kappa }}_{1,B},\cdots ,{\hat{\kappa }}_{L,B}\right)}^{T}=\frac{1}{B}{\left(\sum_{b=1}^{B}{\hat{\kappa }}_{1}^{b},\cdots ,\sum_{b=1}^{B}{\hat{\kappa }}_{L}^{b}\right)}^{T}.$$

The elements (s,t) of *S* can then be determined as follows:

$$S_{ss}=\text{var}\left({\hat{\kappa }}_{s,B}\right)=\frac{\sum_{b=1}^{B}{\left({\hat{\kappa }}_{s}^{b}-{\hat{\kappa }}_{s,B}\right)}^{2}}{B-1},$$

$$S_{st}=\text{cov}\left({\hat{\kappa }}_{s,B},{\hat{\kappa }}_{t,B}\right)=\frac{\sum_{b=1}^{B}\left({\hat{\kappa }}_{s}^{b}-{\hat{\kappa }}_{s,B}\right)\left({\hat{\kappa }}_{t}^{b}-{\hat{\kappa }}_{t,B}\right)}{B-1}\;\;s\neq t$$

The vector ${\hat{\kappa }}_{B}$ and the matrix *S* are then used in Eq. 3.

## 5 Simulations

To study the behavior of the type I error rate (α), we simulated multilevel dependent categorical variables with fixed marginal probability distribution and kappa coefficient between pairs of variables. This was done according to the convex combination algorithm introduced by Lee (1997) and implemented in R by Ibrahim and Suliadi (2011). The algorithm originally considered the coefficient of uncertainty U, Goodman and Kruskal's τ, and Goodman and Kruskal's γ‐coefficient as association measures between pairs of categorical variables. In the present case, these association measures are replaced by Cohen's kappa coefficient when the scale is binary and the linear weighted kappa coefficient when the scale is 3‐ordinal.

Data were simulated for three observers assessing K=20, 30, and 100 clusters with each $n_{k}=2,3$, or 4 items. The kappa coefficient obtained between observers 1 and 2 (namely, κ_1_) was then compared to the kappa coefficient obtained between observers 1 and 3, (namely, κ_2_).

As agreement values, similarly to correlation values, are restricted by the marginal probability distribution of the observers, the three observers were assumed to have the same marginal probability distribution to allow the simulation of interobserver agreement levels between 0 and 1. However, even so, it was not possible to generate data for all the planned simulation patterns. Uniform (0.5,0.5) and nonuniform (0.7,0.3) marginal probability distributions were considered for binary scales while only the uniform (1/3,1/3,1/3) marginal probability distribution was considered in the 3‐ordinal case.

The association structure between the ratings can be divided in three parts: the intracluster association (different items classified by the same observers), the interobserver agreement levels (the same items classified by different observers) and the interobserver association (different items classified by different observers). The association structure was expressed in terms of kappa coefficients in the convex combination algorithm introduced by Lee (1997) instead of the coefficient of uncertainty U, originally used.

The same homogeneous intracluster association structure was considered for each observer. The association strength between members of a cluster, given in terms of kappa coefficients, was fixed to represent no association to strong association within clusters, i.e. $\kappa_{IC}=0,0.1,0.3,0.5$, and 0.7. The interobserver agreement levels for the three pairs of observers were fixed to κ=0, 0.2, 0.4, 0.6, and 0.8 and the interobserver association levels were fixed to values allowed by the algorithm, $\kappa_{dep}=0.3$ and $\kappa_{dep}=0.5$ for the highest interobserver agreement level κ=0.8.

For each simulation scheme, the mean squared error, the mean standard error, the mean correlation between the two agreement coefficients of interest and the type I error, defined as the number of times the Hotelling's *T* ^2^ test rejects the null hypothesis of equal kappa coefficients, were recorded. This was done using the multilevel delta and the clustered bootstrap method for the new multilevel method and when ignoring the multilevel data structure of the data. The clustered bootstrap method was based on B=5000 bootstrap samples. Note that the sample estimate of kappa coefficients is the same either when taking the multilevel data structure into account or not. A total of 500 simulations were performed for each parameter configuration. Therefore, the 95% confidence interval for the type I error is [0.031;0.069].

**Figure 1:**
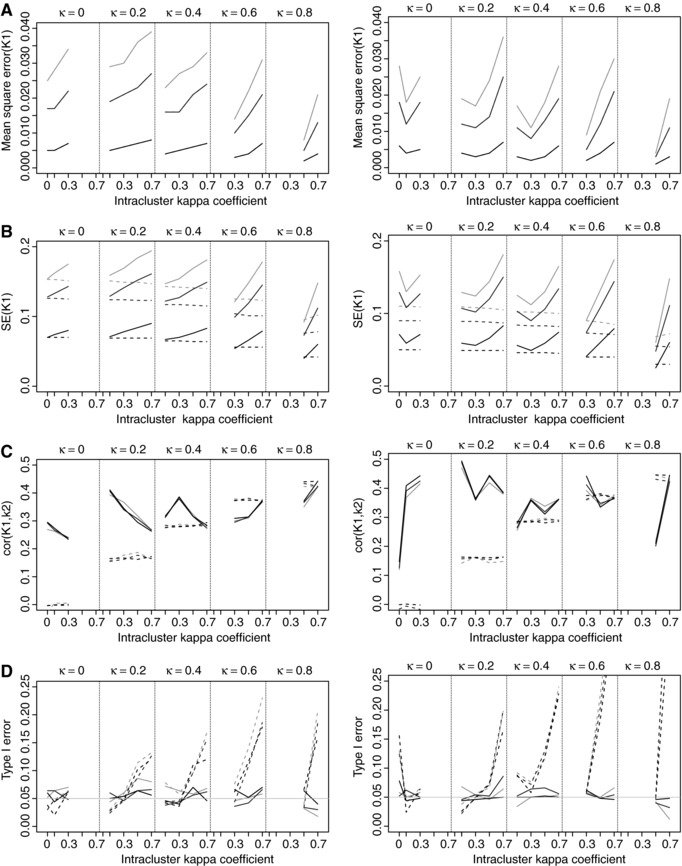
(A) Mean squared error and (B) mean standard error of κ_1_, (C) mean correlation between κ_1_ and κ_2_ and (D) type I error for the comparison of two dependent multilevel kappa coefficients (κ_1_ and κ_2_) obtained on a binary scale when the observers marginal probability distribution is uniform and the cluster size is equal to $n_{k}=2$ (left) and $n_{k}=4$ (right). The results obtained by the delta method ignoring the hierarchical structure (dashed lines) and by the multilevel delta method (plain line) are reported for K=100 (black), K=30 (middle gray), and K=20 (light gray) clusters. Results are depicted for different interobserver agreement values (κ=0,0.2,0.4,0.6,0.8).

The results of the simulations are reported in Fig. 1 under the scenario that the three observers classify items on a binary scale with a uniform marginal probability distribution. Results are only displayed for one of the three kappa coefficients and the delta method because the other results were very similar. The complete results are given in the supporting web material.

As seen in Fig. 1A, the mean squared error of the kappa estimates is relatively small (less than 0.040 for 20 clusters, 0.035 for 30 clusters and 0.010 for 100 clusters) and increases in general with the value of the intra‐cluster kappa coefficient.

When the hierarchical data structure is ignored (dashed lines), the standard error of the kappa coefficients (Fig. 1B) and the correlation between pairs of kappa coefficients (Fig. 1C) does not vary according to the intracluster kappa coefficient. This was to be expected since all items are considered to be independent of each other in that case. When the multilevel structure is accounted for (plain lines), the standard error increases according to the intracluster kappa coefficient. The increase in standard error is roughly equal to the design effect (see e.g. Hox, 2002), that is $D_{eff}=1+\kappa_{IC}*\left(n_{k}-1\right)$ where $\kappa_{IC}$ denotes the intracluster kappa coefficient. This reflects the fact that, when the intracluster kappa coefficient increases, the items of a same cluster become more alike. This decreases the amount of information contained in the data and therefore increases the uncertainty, which is quantified by the standard error. According to the formula given in Appendix 1, the correlation between the kappa coefficients also varies with value of the intracluster kappa coefficient.

The difference in the behavior of the standard error and the correlation between the two types of analysis resulted in different behaviors of the type I error rates (Fig. 1D). The type I error rate increases dramatically outside the 95% confidence interval for intracluster kappa coefficients larger than 0.3. The type I error rate obtained with the multilevel method is closer to the nominal level with a large number of clusters (K=100) than with a small number (K=20), although type I error rates are already within the 95% confidence interval for K=20 in most of the cases.

**Figure 2:**
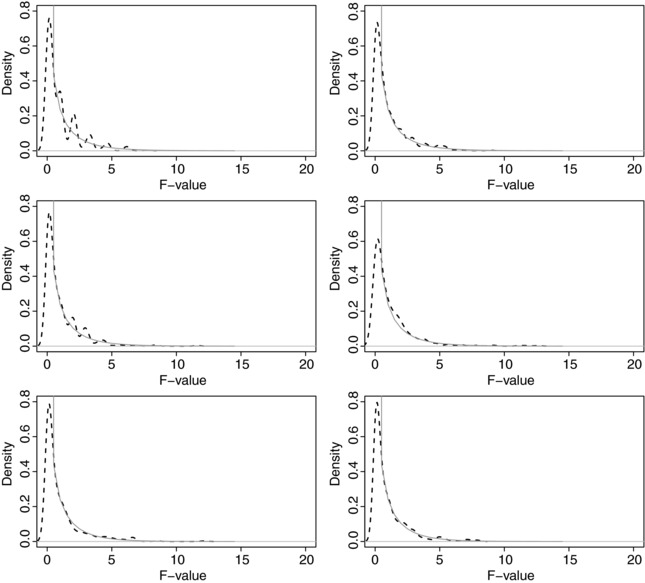
Theoretical (plain line) and observed (dashed line) sampling distribution of the *T* ^2^ statistic when comparing two kappa coefficients equal to 0.8 obtained on a binary scale with uniform observers' marginal distribution when the intracluster kappa coefficient equals 0.5. In the left panel, there are $n_{k}=2$ observations per cluster and in the right panel there are $n_{k}=4$ observations per cluster. The number of clusters is 20 (upper panel), 30 (middle panel) and 100 (lower panel).

In general, the type I error rate is the furthest from the nominal level with the multilevel approach for large interobserver agreement values (κ=0.8) and moderate cluster size (K=20,30). The test also shows somewhat conservative type I error rates for a small number of clusters (K=20) and small cluster size ($n_{k}=2$). One assumption underlying the Hotelling's *T* ^2^ test is the multivariate normality of the kappa coefficients vector. This assumption could be problematic for high agreement values and small sample sizes. Indeed, since kappa coefficients are bounded in the interval [−1,1], the sampling distribution of the kappa coefficients becomes left skewed when approaching the boundaries. To illustrate the effect of this skewness on the sampling distribution of the *T* ^2^ statistic, the density of the 500 *T* ^2^ statistics obtained for κ=0.8 with the intracluster kappa coefficient equal to 0.5 is depicted in Fig. 2 for a binary scale under the uniform marginal distribution of the observers. Some deviations from the theoretical distribution are noted, explaining the behavior of the type I error rate.

## 6 Application

The aim of the third FEES study part (Pilz *et al*., 2016) is to compare the individual intraobserver agreement level of two students to the intraobserver agreement level obtained by the students in consensus. Since the FEES criteria are ordinal, the multilevel linear weighted kappa coefficient is used as agreement measure. Three dependent linear weighted kappa coefficients (observer 1, observer 2, consensus) obtained on multilevel data (two swallows per patients) have therefore to be compared. The criterion “posterior spill” is not analyzed because all observations except two were classified in the category “No”.

The two prerequisites to the definition of a kappa coefficient at the patient level are (1) the absence of patient subpopulations in terms of dysphagia severity and (2) the homogeneity of the dysphagia severity within patient, that is the probability of being classified in the different FEES severity categories should not depend on liquid consistency. There was no evidence against the first assumption in the two first study parts (see Pilz *et al*., 2016). To test the adequacy of the second assumption, the proportion of patients classified in the different FEES severity categories are given in Table 1 according to the liquid consistency. The effect of the consistency on the marginal probability distributions was tested through an ordinal multilevel probit regression. As it can be seen in Table 1, a separate kappa coefficient should be computed per liquid consistency for the valleculae pooling and the penetration/aspiration criteria because there was evidence against the homogeneity assumption.

**Table 1:** FEES study (third part). Proportion of patients classified in the different FEES severity categories according to the liquid consistency (*N*=20). Test of the homogeneity of the dysphagia severity within patient

		Category					
Parametera)	Liquid consistency	1	2	3	4	5	*p*‐valueb)
VP	Thin	0.41	0.55	0.04			<0.0001
	Thick	0.20	0.43	0.37			
PP	Thin	0.57	0.41	0.02			0.41
	Thick	0.65	0.29	0.06			
PD	Thin	0.30	0.22	0.18	0.15	0.15	0.36
	Thick	0.18	0.44	0.18	0.12	0.09	
PA	Thin	0.35	0.60	0.05			<0.0001
	Thick	0.62	0.34	0.04			

^a)^VP, valleculae pooling; PP, pyriform pooling; PD, piecemeal deglutition; PA, penetration/aspiration.

^b)^ *p*‐value obtained by ordinal multilevel probit regression.

The intraobserver agreement level obtained by the students individually and during the consensus meeting are given in Table 2. Because of the small number of observations, an overall linear weighted kappa coefficient was computed for the valleculae pooling and the penetration/aspiration criteria, despite the heterogeneity of the dysphagia severity scoring for the thin and thick liquid consistencies (see Table 2). The agreement coefficients were compared using the delta method and the clustered bootstrap method with B=5000 iterations. The multilevel structure of the data was taken into account when the agreement was computed at patient level. Note that the program took less than 1 s for the multilevel delta method and about 16 s for the clustered bootstrap method on a regular PC (Intel Core II, 2GB).

**Table 2:** FEES study. Intraobserver agreement level (linear weighted kappa coefficient and standard errors obtained with the multilevel delta method and the clustered bootstrap method) for the 4 FEES variables. The *p*‐value refers to the comparison of the three multilevel dependent kappa coefficients

			Delta method				
Parametera)	Kb)	Nc)	Liquid consistencyd)	Observer 1	Observer 2	Consensus	p‐value
VP	14	20	All	0.79 (0.11)	0.94 (0.061)	0.75 (0.12)	0.25
	14	14	Thin	0.69 (0.21)	1.00 (NA)	0.66 (0.18)	NA
	6	6	Thick	0.57 (0.39)	0.57 (0.39)	0.79 (0.21)	NA
PP	19	29	All	0.56 (0.19)	0.76 (0.11)	1.00 (NA)	NA
PD	20	35	All	0.93 (0.037)	0.78 (0.081)	0.94 (0.034)	0.11
PA	18	26	All	0.62 (0.14)	0.79 (0.098)	0.88 (0.071)	0.25
	13	13	Thin	0.84 (0.15)	0.48 (0.23)	0.80 (0.12)	0.28
	13	13	Thick	0.35 (0.28)	1.00 (NA)	1.00 (NA)	NA
			Clustered bootstrap method				
Parameter	K	N	Liquid consistency	Observer 1	Observer 2	Consensus	p‐value
VP	20	40	All	0.74 (0.12)	0.94 (0.053)	0.84 (0.074)	0.13
	20	20	Thin	0.55 (0.23)	1.00 (NA)	0.71 (0.15)	NA
	20	20	Thick	0.72 (0.29)	0.82 (0.18)	0.92 (0.080)	0.62
PP	20	40	All	0.54 (0.18)	0.75 (0.12)	0.90 (0.075)	0.13
PD	20	40	All	0.94 (0.036)	0.76 (0.088)	0.95 (0.030)	0.10
PA	20	40	All	0.64 (0.13)	0.81 (0.088)	0.93 (0.049)	0.14
	20	20	Thin	0.84 (0.15)	0.58 (0.21)	0.85 (0.092)	0.41
	20	20	Thick	0.40 (0.25)	1.00 (NA)	1.00 (NA)	NA

^a)^VP, valleculae pooling; PP, pyriform pooling; PD, piecemeal deglutition; PA, penetration/aspiration.

^b)^K is the number of patients.

^c)^N is the total number of observations.

^d)^A separate kappa coefficient was computed for each liquid consistency when dysphagia scores were different for thin and thick liquids.

The results from the multilevel delta and the clustered bootstrap methods differ, mainly because they are based on a different number of observations. In fact, by definition of the methods, the delta method is based on complete cases analysis while the clustered bootstrap method uses available cases. When considering only the complete cases, parameter estimates and the *p*‐values obtained with the clustered bootstrap method are closer to what is obtained with the delta method (i.e., the *p*‐values are 0.24 for VP, NA for PP, 0.11 for PD, and 0.24 for PA).

All the agreement coefficients were positive with minimum and maximum agreement values both obtained for pyriform pooling (0.56 and 1.00). Observer 1 showed the largest variability in agreement values (range: 0.56–0.93). There was no evidence of a difference in the intraobserver agreement levels obtained individually and in consensus. This suggests that consensus ratings might offer an alternative to independent rating of FEES exams. However, changes in the scoring of the FEES criteria between the individual and the consensus ratings were observed (data not shown, Pilz *et al*., 2016). Therefore, the validity of the FEES criteria for individual and consensus ratings also needs to be studied in order to better compare the two rating processes. It is worth noting that the conclusions of the study should be taken with great care because of the small sample size of this exploratory study.

**Figure 3:**
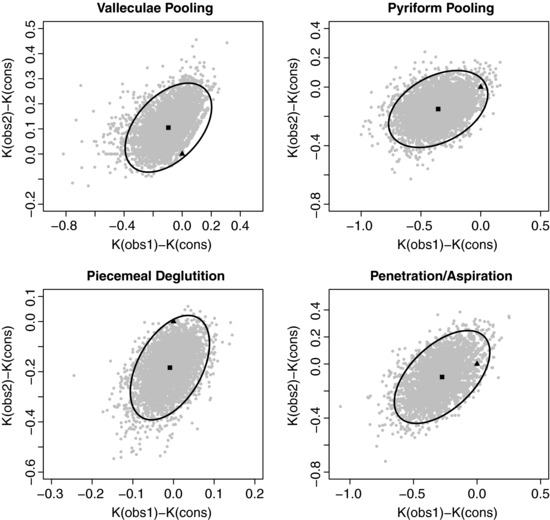
Differences between the kappa coefficients obtained by the observers individually and in consensus with the clustered bootstrap method (95% confidence ellipse). The square represents the bootstrap estimate and the triangle the origin point (0,0).

The 5000 differences between the pairs of kappa coefficients generated by the clustered bootstrap method are depicted in Fig. 3 with 95% confidence ellipse. As expected from the results in Table 2, the point (0,0) lies in the confidence ellipse for the four FEES variables. Note that the bootstrap estimates can show some unexpected pattern (e.g. regions in the 95% confidence ellipse almost empty) because the marginal probability distribution of the observers limits the possible values of kappa coefficients. These patterns, if present, could directly challenge the multivariate normality assumption of the kappa coefficients vector. This is however not the case here where the bootstrap estimates are harmoniously distributed in the 95% confidence ellipse for the four FEES variables.

## 7 Discussion

A simple method based on the use of Hotelling's *T* ^2^ statistic was developed in this paper to compare dependent kappa coefficients obtained on multilevel data, a frequent situation in medical research. This method can easily be implemented in practice because it is based on simple matrix calculations. A R package “multiagree” was developed by the author and is available on github. The code to reproduce the results presented in this paper and install the package is available as Supporting Information on the journal's web page (http://onlinelibrary.wiley.com/doi/bimj.201600093/suppinfo). Additionally to the methods presented in this paper, this package also considers the case of several observers, independent kappa coefficients and kappa coefficients obtained on independent observations. The method of Fleiss (1981) (cfr Appendix 2) can be used to compare independent kappa coefficients (or other measures) by using standard errors derived with the multilevel delta or the clustered bootstrap method. The package can be used for all multilevel studies where two or more kappa coefficients have to be compared. In contrast, modeling techniques require more specific programming skills and a new program has to be written for each specific study. Nevertheless, their use is highly recommended in the presence of several covariates or of continuous covariates.

Two assumptions were made by Yang and Zhou (2014) to ensure the existence of an overall kappa coefficient, that is the homogeneity of the members of a cluster and the existence of a common kappa coefficient across the clusters. When there is evidence that the assumptions do not hold, as discussed by Yang and Zhou (2014), a separate kappa coefficient should be computed for each subpopulation identified.

A third assumption was necessary to ensure that the sampling distribution of the *T* ^2^ statistic is a F‐distribution, that is the multivariate normality of the vector of kappa coefficients. When the sample size is large, a normal sampling distribution of the kappa coefficients is ensured by the central limit theorem. However, normality could be problematic for small sample sizes (*K*=20) and large kappa values (κ=0.8), as discussed in the simulation section. This was however not the case in the FEES study with only K=20 patients involved (see Fig. 3). The use of nonparametric alternatives to the *T* ^2^ statistic to compare dependent kappa coefficients is a topic for future research.

Accounting for the hierarchical structure of the data is strongly advised, even for small numbers of clusters and small cluster sizes, as shown in the simulations. Ignoring the hierarchical structure of the data can in general increase dramatically the type I error rate for intracluster kappa values above 0.3. These results are consistent with the results of Yang and Zhou (2014), where a good performance of the multilevel delta method was observed for small number of clusters (e.g. K=25) and moderate cluster sizes (e.g. $n_{k}\leq 10$). These conclusions should however be taken with caution due to the limited simulation schemes considered in this paper.

The multilevel delta method, although asymptotic, showed similar coverage levels than the clustered bootstrap method. However, in the presence of missing data, the use of the delta and the clustered bootstrap methods can lead to different conclusions because the delta method, by definition, is based on a complete case analysis while the clustered bootstrap method is based on an available case analysis. If data are not missing completely at random, both analyses may give bias estimates and invalid inference. Likelihood‐based methods could then be preferred. When the amount of missing data is important, using the multilevel delta method can reduce the sample size drastically, as for the valleculae pooling criterion in the FEES study and lead to inefficient analysis. The clustered bootstrap method is less affected. One other advantage of the clustered bootstrap method over the multilevel delta method is its simplicity. It can easily extend to other measures (e.g., agreement between several observers, price delay (Bae *et al*., 2012) in finance) while specific mathematical derivations are required for each new statistical measure considered to compute the variance‐covariance matrix with the delta method.

To summarize, this paper provides a simple method to compare dependent agreement measures obtained on multilevel data and performs well even when the number of clusters is small (K=20). The method should however be used with care when both the number of clusters and the number of observations per clusters are small. This method can be easily extended to other measures if the clustered bootstrap method is used to compute the variance‐covariance matrix. However, modeling techniques are highly recommended in the presence of several or continuous covariates. Likewise, the used of likelihood based techniques might be preferable if the amount of missing data is important.

## Conflict of interest

*The authors have declared no conflict of interest*.

## Supporting information

Comparing dependent kappa coefficients obtained on multilevel data Supplementary material.

Supporting Information.

## Appendix A

### A.1 Derivation of the variance‐covariance matrix

Denote the vector containing the agreement observed in each cluster by $P_{ol}=\left(P_{ol,1},\cdots ,P_{ol,K}\right)$ (l=1,2), the vector with the weight relative to each cluster by $\nu ={\left(\nu_{1},\cdots ,\nu_{K}\right)}^{T}$, the matrix with the *K* cluster‐specific marginal classification distributions by $p_{•+++,•}={\left(p_{•+++,1},\cdots ,p_{•+++,K}\right)}_{g\times K}$, $p_{+•++,•}={\left(p_{+•++,1},\cdots ,p_{+•++,K}\right)}_{g\times K}$, $p_{++•+,•}={\left(p_{++•+,1},\cdots ,p_{++•+,K}\right)}_{g\times K}$, and $p_{+++•,•}={\left(p_{+++•,1},\cdots ,p_{+++•,K}\right)}_{g\times K}$ for observer 1, 2, 3, and 4, respectively.

Using these notations, the vector with the overall marginal classification distribution for the four observers are given by $p_{•+++}=p_{•+++,•}\nu$, $p_{+•++}=p_{+•++,•}\nu$, $p_{++•+}=p_{++•+,•}\nu$ and $p_{+++•}=p_{+++•,•}\nu$. Further let $\Omega =\left(I-\nu 1_{K\times 1}^{T}\right)\text{diag}\left(\nu_{1}^{2},\cdots ,\nu_{K}^{2}\right)\left(I-1_{K\times 1}\nu^{T}\right)$. Similarly to Yang and Zhou (2014), it can be shown that under mild regular conditions, the vector $\hat{\xi }$ is asymptotically normally distributed with variance‐covariance matrix given by

$$\text{var}\left(\hat{\xi }\right)=\frac{1}{K}\left(\begin{array}{cccccc} V_{11} & \;V_{12} & \;V_{13} & \;V_{14} & \;V_{15} & \;V_{16}\\ V_{21} & \;V_{22} & \;V_{23} & \;V_{24} & \;V_{25} & \;V_{26}\\ V_{31} & \;V_{32} & \;V_{33} & \;V_{34} & \;V_{35} & \;V_{36}\\ V_{41} & \;V_{42} & \;V_{43} & \;V_{44} & \;V_{45} & \;V_{46}\\ V_{51} & \;V_{52} & \;V_{53} & \;V_{54} & \;V_{55} & \;V_{56}\\ V_{61} & \;V_{62} & \;V_{63} & \;V_{64} & \;V_{65} & \;V_{66} \end{array}\right).$$

The elements of $\text{var}(\hat{\xi })$ can be estimated following the techniques of Rao and Scott (1992) and Obuchowski (1998). The variances and the covariance between the observed agreement are given by

$$\begin{array}{cc} {\hat{V}}_{ll}=\frac{K^{2}}{K-1}P_{ol}\Omega P_{ol}^{T},\;\;\left(l=1,2\right)\;\;\text{and} & {\hat{V}}_{12}={\hat{V}}_{21}=\frac{K^{2}}{K-1}P_{o1}\Omega P_{o2}^{T},\;\text{respectively.} \end{array}$$

The variance‐covariance part relative to the observed agreement and the marginal probability distribution of the four observers is given by

$$\begin{array}{cc} {\hat{V}}_{13}={\hat{V}}_{31}^{T}=\frac{K^{2}}{K-1}P_{o1}\Omega p_{•+++,•}^{T} & {\hat{V}}_{14}={\hat{V}}_{41}^{T}=\frac{K^{2}}{K-1}P_{o1}\Omega p_{+•++,•}^{T}\\ {\hat{V}}_{15}={\hat{V}}_{51}^{T}=\frac{K^{2}}{K-1}P_{o1}\Omega p_{++•+,•}^{T} & {\hat{V}}_{16}={\hat{V}}_{61}^{T}=\frac{K^{2}}{K-1}P_{o1}\Omega p_{+++•,•}^{T} \end{array}$$

for the observed agreement between observers 1 and 2 and by

$$\begin{array}{cc} {\hat{V}}_{23}={\hat{V}}_{32}^{T}=\frac{K^{2}}{K-1}P_{o2}\Omega p_{•+++,•}^{T} & {\hat{V}}_{24}={\hat{V}}_{42}^{T}=\frac{K^{2}}{K-1}P_{o2}\Omega p_{+•++,•}^{T}\\ {\hat{V}}_{25}={\hat{V}}_{52}^{T}=\frac{K^{2}}{K-1}P_{o2}\Omega p_{++•+,•}^{T} & {\hat{V}}_{26}={\hat{V}}_{62}^{T}=\frac{K^{2}}{K-1}P_{o2}\Omega p_{+++•,•}^{T} \end{array}$$

for the observed agreement between observers 3 and 4. Finally, the variance‐covariance part between the marginal probabilities distribution of the four observers is given by

$$\begin{array}{cc} {\hat{V}}_{33}=\frac{K^{2}}{K-1}p_{•+++,•}\Omega p_{•+++,•}^{T}, & {\hat{V}}_{34}=\frac{K^{2}}{K-1}p_{•+++,•}\Omega p_{+•++,•}^{T}\\ {\hat{V}}_{35}=\frac{K^{2}}{K-1}p_{•+++,•}\Omega p_{++•+,•}^{T} & {\hat{V}}_{36}=\frac{K^{2}}{K-1}p_{•+++,•}\Omega p_{+++•,•}^{T}\\ {\hat{V}}_{44}=\frac{K^{2}}{K-1}p_{+•++,•}\Omega p_{+•++,•}^{T}, & {\hat{V}}_{45}=\frac{K^{2}}{K-1}p_{+•++,•}\Omega p_{++•+,•}^{T}\\ {\hat{V}}_{46}=\frac{K^{2}}{K-1}p_{+•++,•}\Omega p_{+++•,•}^{T} & {\hat{V}}_{55}=\frac{K^{2}}{K-1}p_{++•+,•}\Omega p_{++•+,•}^{T},\\ {\hat{V}}_{56}=\frac{K^{2}}{K-1}p_{++•+,•}\Omega p_{+++•,•}^{T} & {\hat{V}}_{66}=\frac{K^{2}}{K-1}p_{+++•,•}\Omega p_{+++•,•}^{T}. \end{array}$$

Two applications of the delta method give the variance‐covariance matrix $\hat{S}$ for the vector of kappa coefficients $\hat{\kappa }={\left({\hat{\kappa }}_{1},{\hat{\kappa }}_{2}\right)}^{T}$,

$$\begin{array}{ccc} cov({\hat{\kappa }}_{1},{\hat{\kappa }}_{2}) & = & \frac{{\hat{V}}_{12}}{\left(1-P_{e1}\right)\left(1-P_{e2}\right)}+\frac{\left(P_{o1}-1\right)}{{\left(1-P_{e1}\right)}^{2}\left(1-P_{e2}\right)}\left(p_{+•++}^{T}\Omega^{T}{\hat{V}}_{32}+p_{•+++}^{T}\Omega {\hat{V}}_{42}\right)+\\ & & +\frac{\left(P_{o1}-1\right)\left(P_{o2}-1\right)}{{\left(1-P_{e1}\right)}^{2}{\left(1-P_{e2}\right)}^{2}}\left(p_{+•++}^{T}\Omega^{T}{\hat{V}}_{35}p_{+++•}^{T}\Omega^{T}+p_{+•++}^{T}\Omega^{T}{\hat{V}}_{36}p_{++•+}^{T}\Omega \right.+\\ & + & \left.p_{•+++}^{T}\Omega {\hat{V}}_{45}p_{+++•}^{T}\Omega^{T}+p_{•+++}^{T}\Omega {\hat{V}}_{46}p_{++•+}^{T}\Omega \right)+\\ & + & \frac{\left(P_{o2}-1\right)}{\left(1-P_{e1}\right){\left(1-P_{e2}\right)}^{2}}\left({\hat{V}}_{15}p_{+++•}^{T}\Omega^{T}+{\hat{V}}_{16}p_{++•+}\Omega \right) \end{array}$$

$$\begin{array}{ccc} var({\hat{\kappa }}_{1}) & = & \frac{{\hat{V}}_{11}}{{\left(1-P_{e1}\right)}^{2}}+\frac{\left(P_{o1}-1\right)}{{\left(1-P_{e1}\right)}^{3}}\left({\hat{V}}_{13}p_{+•++}^{T}\Omega^{T}+{\hat{V}}_{14}p_{•+++}^{T}\Omega +p_{+•++}^{T}\Omega^{T}{\hat{V}}_{31}+p_{•+++}^{T}\Omega {\hat{V}}_{41}\right)+\\ & & +\frac{{\left(P_{o1}-1\right)}^{2}}{{\left(1-P_{e1}\right)}^{4}}\left(p_{+•++}^{T}\Omega^{T}{\hat{V}}_{33}p_{+•++}^{T}\Omega^{T}+p_{•+++}^{T}\Omega {\hat{V}}_{44}p_{•+++}^{T}\Omega \right.+\\ & & +\left.p_{+•++}^{T}\Omega^{T}{\hat{V}}_{34}p_{•+++}^{T}\Omega +p_{•+++}^{T}\Omega {\hat{V}}_{43}p_{+•++}^{T}\Omega^{T}\right) \end{array}$$

$$\begin{array}{ccc} var({\hat{\kappa }}_{2}) & = & \frac{{\hat{V}}_{22}}{{\left(1-P_{e2}\right)}^{2}}+\frac{\left(P_{o2}-1\right)}{{\left(1-P_{e2}\right)}^{3}}\left({\hat{V}}_{25}p_{+++•}^{T}\Omega^{T}+{\hat{V}}_{26}p_{++•+}^{T}\Omega +p_{+++•}^{T}\Omega^{T}{\hat{V}}_{52}+p_{++•+}^{T}\Omega {\hat{V}}_{62}\right)+\\ & & +\frac{{\left(P_{o2}-1\right)}^{2}}{{\left(1-P_{e2}\right)}^{4}}\left(p_{+++•}^{T}\Omega^{T}{\hat{V}}_{55}p_{+++•}^{T}\Omega^{T}+p_{++•+}^{T}\Omega {\hat{V}}_{66}p_{++•+}^{T}\Omega \right.+\\ & & +\left.p_{+++•}^{T}\Omega^{T}{\hat{V}}_{56}p_{++•+}^{T}\Omega +p_{++•+}^{T}\Omega {\hat{V}}_{65}p_{+++•}^{T}\Omega^{T}\right) \end{array}$$

### A.2 Comparison of independent kappa coefficients

To compare association coefficients, Fleiss (1981) developed a method directly inspired by the classical one‐way analysis of variance and the chi‐square decomposition theory. This methodology can be applied to kappa coefficients. Consider *L* independent estimates of a kappa coefficient (${\hat{\kappa }}_{1},\cdots ,{\hat{\kappa }}_{L}$). The coefficient ${\hat{\kappa }}_{l}$ denotes the kappa coefficient relative to modality *l* of the categorical covariate (l=1,⋯,L). Let $\text{SE}({\hat{\kappa }}_{l})$ be the standard error of the kappa coefficient ${\hat{\kappa }}_{l}$ and $w_{l}=1/{\left[\text{SE}\left({\hat{\kappa }}_{l}\right)\right]}^{2}$. In the presence of multilevel data, the standard error can be obtained using the methods presented in Section 4. Under the hypothesis of agreement only due to chance in the modality *l* of the covariate, the statistic

$$\chi_{l}=\frac{{\hat{\kappa }}_{l}}{\text{SE}({\hat{\kappa }}_{l})}={\hat{\kappa }}_{l}\sqrt{w_{l}}$$

approximately follows a normal distribution (Central‐Limit theorem) and the statistic $\chi_{l}^{2}=w_{l}{\hat{\kappa }}_{l}^{2}$ approximately follows a chi‐square distribution with one degree of freedom if the sample sizes $n_{l}$ (l=1,⋯,L) are sufficiently “large.” Fleiss (1981) considered the statistic $\chi_{tot}^{2}=\sum_{l=1}^{L}\chi_{l}^{2}$ to compare the *L* kappa coefficients and divided $\chi_{tot}^{2}$ in two terms $\chi_{tot}^{2}=\chi_{hom}^{2}+\chi_{ass}^{2}$ where $\chi_{hom}^{2}$ represents the homogeneity degree between the *L* kappa coefficients and $\chi_{ass}^{2}$ represents a mean degree of agreement. The term $\chi_{ass}^{2}$ is computed as follows,

$${\hat{\kappa }}_{ass}=\frac{\sum_{l=1}^{L}w_{l}{\hat{\kappa }}_{l}}{\sum_{l=1}^{L}w_{l}}.$$

Under the hypothesis of a global kappa coefficient equal to 0, ${\hat{\kappa }}_{ass}$ has a value of 0 and $\text{SE}\left({\hat{\kappa }}_{ass}\right)=1/\sqrt{\sum_{l=1}^{L}w_{l}}$. The statistic $Z_{ass}={\hat{\kappa }}_{ass}/SE\left({\hat{\kappa }}_{ass}\right)$ is thus normally distributed. Then,

$$\chi_{ass}^{2}=Z_{ass}^{2}={\hat{\kappa }}_{ass}^{2}\sum_{l=1}^{L}w_{l}=\frac{{\left(\sum_{l=1}^{L}w_{l}{\hat{\kappa }}_{l}\right)}^{2}}{\sum_{l=1}^{L}w_{l}}$$

approximately follows a chi‐square distribution with one degree of freedom. The term $\chi_{hom}^{2}$ is obtained by subtraction.

$$\chi_{hom}^{2}=\chi_{tot}^{2}-\chi_{ass}^{2}=\sum_{l=1}^{L}w_{l}{\hat{\kappa }}_{l}^{2}-{\hat{\kappa }}_{ass}^{2}\sum_{l=1}^{L}w_{l}=\sum_{l=1}^{L}\frac{{\left({\hat{\kappa }}_{l}-{\hat{\kappa }}_{ass}\right)}^{2}}{\text{SE}{\left({\hat{\kappa }}_{l}\right)}^{2}}.$$

In order to test the hypothesis $H_{0}:\;\kappa_{1}=\cdots =\kappa_{L}\;\;\text{vs}\;\;H_{1}:\;\exists \;l\neq m\;:\;\kappa_{l}\neq \kappa_{m}\;\left(l,m\in \left\{1,\cdots ,L\right\}\right)$, we have to compare $\chi_{hom}^{2}$ to the chi‐square distribution with L−1 degrees of freedom, the null hypothesis being rejected at the α confidence level if $\chi_{hom}^{2}$ is greater than $Q_{\chi^{2}}\left(1-\alpha ;L-1\right)$, the (1−α)‐quantile of the chi‐square distribution on L−1 degrees of freedom.
