# Supplementary material for: Comparing dependent kappa coefficients obtained on multilevel data
Source: Biom J. 2017 May 2;59(5):1016–34. doi: 10.1002/bimj.201600093 (PMC5600130; doi:10.1002/bimj.201600093)
Supplement: Supplementary file 1 — Comparing dependent kappa coefficients obtained on multilevel data Supplementary material. [file BIMJ-59-1016-s001.pdf]

Comparing dependent kappa coefficients obtained on multilevel data
Supplementary material

Sophie Vanbelle
Department of Methodology and Statistics, CAPHRI, Maastricht University, The Netherlands

1 Additional simulation results

1.1 Binary scale - uniform marginal distributions

The delta method ignoring the hierarchical structure is compared to the multilevel delta method in Table 1 and to the clustered bootstrap method in Table 2 for a binary scale with uniform marginal distributions when there are two observations per cluster. When there are four observations per cluster, the tables are respectively Table 3 and Table 4. The column K refers to the number of clusters, the column κ to the theoretical value of the two kappa coefficients and the column INT to the intra-cluster kappa level. Then, the mean value over the 500 simulations of the two kappa coefficients to be compared is given along with the mean standard error for each method. The column COR refers to the mean correlation between the two kappa coefficients to be compared. Its theoretical value was 0.3, except when $\kappa = 0.8$ where it was fixed to 0.5. Lastly, the column α provide the estimated type I error. With 500 simulations, a 95% confidence interval for the nominal level is 0.031-0.069. Values outside this interval are indicated in light gray. The results from the simulation are also depicted in Figure 1.

1.2 Binary scale - marginal distribution (0.7,0.3)

The delta method ignoring the hierarchical structure is compared to the multilevel delta method in Table 5 and to the clustered bootstrap method in Table 6 for a binary scale with uniform marginal distributions when there are two observations per cluster. When there are four observations per cluster, the tables are respectively Table 7 and Table 8. The column K refers to the number of clusters, the column κ to the theoretical value of the two kappa coefficients and the column INT to the intra-cluster kappa level. Then, the mean value over the 500 simulations of the two kappa coefficients to be compared is given along with the mean standard error for each method. The column COR refers to the mean correlation between the two kappa coefficients to be compared. Its theoretical value was 0.3, except when $\kappa = 0.8$ where it was fixed to 0.5. Lastly, the column α provide the estimated type I error. With 500 simulations, a 95% confidence interval for the nominal level is 0.031-0.069. Values outside this interval are indicated in light gray. The results from the simulation are also depicted in Figure 2.

1.3 Three-ordinal scale - uniform marginal distributions

The delta method ignoring the hierarchical structure is compared to the multilevel delta method in Table 9 and to the clustered bootstrap method in Table 10 for a 3-ordinal scale with uniform marginal distributions when there are two observations per cluster. When there are three observations per cluster, the tables are respectively Table 11 and Table 12. The column K refers to the number of clusters, the column κ to the theoretical value of the two kappa coefficients and the column INT to the intra-cluster kappa level. Then, the mean value over the 500 simulations of the two kappa coefficients to be compared is given along with the mean standard error for each method. The column COR refers to the mean correlation between the two kappa coefficients to be compared. Its theoretical value was 0.3, except when $\kappa = 0.8$ where it was fixed to 0.5. Lastly, the column α provide the estimated type I error. With 500 simulations, a 95% confidence interval for the nominal level is 0.031-0.069. Values outside this interval are indicated in light gray. The results from the simulation are also depicted in Figure 3.
The delta method ignoring the hierarchical structure is compared to the multilevel delta method in Table 9 and to the clustered bootstrap method in Table 10 for a 3-ordinal scale with uniform marginal distributions when there are two observations per cluster. When there are three observations per cluster, the tables are respectively Table 11 and Table 12. The column K refers to the number of clusters, the column κ to the theoretical value of the two kappa coefficients and the column INT to the intra-cluster kappa level. Then, the mean value over the 500 simulations of the two kappa coefficients to be compared is given along with the mean standard error for each method. The column COR refers to the mean correlation between the two kappa coefficients to be compared. Its theoretical value was 0.3, except when $\kappa = 0.8$ where it was fixed to 0.5. Lastly, the column α provide the

estimated type I error. With 500 simulations, a 95% confidence interval for the nominal level is 0.031-0.069. Values outside this interval are indicated in light gray. The results from the simulation are also depicted in Figure 3.

Table 1: Simulation results obtained for a binary scale with uniform marginal distributions for $n_k = 2$ (Delta method)

Parameters					Delta method - classic				Delta method - multilevel			
K	κ	INT	κ_1	κ_2	$SE(\kappa_1)$	$SE(\kappa_2)$	$CORR$	α	$SE(\kappa_1)$	$SE(\kappa_2)$	$CORR$	α
20	0.0	0.0	0.006	0.005	0.153	0.153	-0.026	0.030	0.154	0.154	0.269	0.052
20	0.0	0.1	0.000	-0.001	0.153	0.153	0.005	0.048	0.162	0.163	0.263	0.064
20	0.0	0.3	-0.009	-0.008	0.151	0.152	0.005	0.064	0.175	0.174	0.236	0.070
20	0.2	0.1	0.191	0.177	0.151	0.151	0.157	0.030	0.159	0.158	0.393	0.044
20	0.2	0.3	0.195	0.191	0.149	0.150	0.177	0.058	0.169	0.171	0.367	0.060
20	0.2	0.5	0.186	0.194	0.149	0.149	0.187	0.114	0.184	0.183	0.318	0.086
20	0.2	0.7	0.189	0.201	0.147	0.147	0.163	0.132	0.194	0.194	0.267	0.080
20	0.4	0.1	0.392	0.385	0.143	0.143	0.274	0.078	0.147	0.149	0.312	0.072
20	0.4	0.3	0.385	0.395	0.142	0.142	0.288	0.058	0.154	0.154	0.383	0.064
20	0.4	0.5	0.388	0.379	0.141	0.141	0.285	0.096	0.169	0.169	0.323	0.058
20	0.4	0.7	0.382	0.388	0.140	0.139	0.284	0.168	0.181	0.181	0.274	0.068
20	0.6	0.3	0.591	0.584	0.125	0.126	0.379	0.076	0.121	0.121	0.300	0.046
20	0.6	0.5	0.578	0.591	0.125	0.124	0.374	0.140	0.148	0.146	0.310	0.052
20	0.6	0.7	0.579	0.576	0.123	0.123	0.377	0.230	0.178	0.178	0.375	0.058
20	0.8	0.5	0.790	0.787	0.095	0.096	0.423	0.040	0.092	0.092	0.350	0.032
20	0.8	0.7	0.741	0.745	0.102	0.102	0.423	0.208	0.148	0.148	0.422	0.018
30	0.0	0.0	-0.001	-0.014	0.126	0.126	-0.003	0.030	0.128	0.128	0.292	0.064
30	0.0	0.1	0.003	-0.006	0.126	0.126	-0.002	0.048	0.133	0.134	0.267	0.064
30	0.0	0.3	-0.003	0.004	0.125	0.125	-0.003	0.052	0.143	0.144	0.240	0.048
30	0.2	0.1	0.196	0.201	0.124	0.124	0.155	0.024	0.129	0.129	0.406	0.060
30	0.2	0.3	0.189	0.192	0.124	0.124	0.162	0.046	0.141	0.141	0.340	0.046
30	0.2	0.5	0.185	0.193	0.123	0.123	0.177	0.088	0.152	0.151	0.307	0.064
30	0.2	0.7	0.186	0.184	0.123	0.123	0.165	0.126	0.161	0.161	0.268	0.066
30	0.4	0.1	0.391	0.392	0.117	0.117	0.284	0.044	0.122	0.121	0.318	0.038
30	0.4	0.3	0.392	0.394	0.117	0.117	0.281	0.046	0.127	0.127	0.379	0.044
30	0.4	0.5	0.384	0.382	0.116	0.117	0.283	0.096	0.139	0.139	0.316	0.056
30	0.4	0.7	0.389	0.392	0.115	0.115	0.282	0.156	0.149	0.149	0.274	0.062
30	0.6	0.3	0.585	0.597	0.104	0.103	0.372	0.044	0.099	0.098	0.295	0.036
30	0.6	0.5	0.598	0.595	0.101	0.102	0.381	0.108	0.119	0.120	0.314	0.042
30	0.6	0.7	0.586	0.590	0.101	0.101	0.370	0.186	0.145	0.145	0.368	0.066
30	0.8	0.5	0.804	0.799	0.076	0.077	0.434	0.034	0.073	0.073	0.366	0.034
30	0.8	0.7	0.780	0.782	0.078	0.078	0.424	0.186	0.112	0.112	0.423	0.030
100	0.0	0.0	0.005	-0.001	0.070	0.070	-0.004	0.038	0.070	0.070	0.295	0.060
100	0.0	0.1	-0.004	-0.003	0.070	0.070	-0.001	0.020	0.074	0.074	0.274	0.044
100	0.0	0.3	-0.005	-0.004	0.070	0.070	0.001	0.062	0.080	0.080	0.234	0.062
100	0.2	0.1	0.195	0.199	0.069	0.069	0.165	0.028	0.071	0.071	0.411	0.050
100	0.2	0.3	0.201	0.204	0.069	0.069	0.167	0.048	0.078	0.078	0.345	0.054
100	0.2	0.5	0.201	0.196	0.069	0.069	0.162	0.102	0.084	0.084	0.295	0.064
100	0.2	0.7	0.193	0.202	0.069	0.069	0.172	0.120	0.090	0.090	0.263	0.056
100	0.4	0.1	0.397	0.397	0.065	0.065	0.277	0.042	0.067	0.067	0.313	0.046
100	0.4	0.3	0.397	0.394	0.065	0.065	0.279	0.036	0.070	0.070	0.386	0.040
100	0.4	0.5	0.397	0.404	0.064	0.064	0.281	0.110	0.076	0.076	0.316	0.070
100	0.4	0.7	0.398	0.403	0.064	0.064	0.294	0.120	0.083	0.082	0.284	0.046
100	0.6	0.3	0.599	0.603	0.056	0.056	0.374	0.062	0.054	0.054	0.309	0.066
100	0.6	0.5	0.599	0.598	0.056	0.056	0.374	0.116	0.066	0.066	0.314	0.052
100	0.6	0.7	0.601	0.595	0.056	0.056	0.373	0.178	0.079	0.080	0.372	0.070
100	0.8	0.5	0.800	0.799	0.042	0.042	0.440	0.066	0.040	0.040	0.371	0.064
100	0.8	0.7	0.796	0.798	0.042	0.042	0.442	0.160	0.060	0.006	0.442	0.040

Table 2: Simulation results obtained for a binary scale with uniform marginal distributions for $n_k = 2$ (Bootstrap method)

Parameters					Delta method - classic				Bootstrap method - multilevel			
K	κ	INT	κ_1	κ_2	$SE(\kappa_1)$	$SE(\kappa_2)$	$CORR$	α	$SE(\kappa_1)$	$SE(\kappa_2)$	$CORR$	α
20	0.0	0.0	0.006	0.005	0.153	0.153	-0.026	0.030	0.151	0.151	0.266	0.050
20	0.0	0.1	0.000	-0.001	0.153	0.153	0.005	0.048	0.158	0.159	0.263	0.066
20	0.0	0.3	-0.009	-0.008	0.151	0.152	0.005	0.064	0.169	0.169	0.239	0.070
20	0.2	0.1	0.191	0.177	0.151	0.151	0.157	0.030	0.156	0.156	0.392	0.048
20	0.2	0.3	0.195	0.191	0.149	0.15	0.177	0.058	0.166	0.167	0.371	0.064
20	0.2	0.5	0.186	0.194	0.149	0.149	0.187	0.114	0.179	0.178	0.323	0.088
20	0.2	0.7	0.189	0.201	0.147	0.147	0.163	0.132	0.188	0.189	0.273	0.078
20	0.4	0.1	0.392	0.385	0.143	0.143	0.274	0.078	0.146	0.148	0.309	0.074
20	0.4	0.3	0.385	0.395	0.142	0.142	0.288	0.058	0.153	0.152	0.383	0.072
20	0.4	0.5	0.388	0.379	0.141	0.141	0.285	0.096	0.166	0.166	0.326	0.062
20	0.4	0.7	0.382	0.388	0.14	0.139	0.284	0.168	0.177	0.177	0.277	0.076
20	0.6	0.3	0.591	0.584	0.125	0.126	0.379	0.076	0.122	0.122	0.296	0.052
20	0.6	0.5	0.578	0.591	0.125	0.124	0.374	0.140	0.147	0.145	0.312	0.066
20	0.6	0.7	0.579	0.576	0.123	0.123	0.377	0.230	0.175	0.175	0.381	0.058
20	0.8	0.5	0.790	0.787	0.095	0.096	0.423	0.040	0.094	0.094	0.346	0.038
20	0.8	0.7	0.741	0.745	0.102	0.102	0.423	0.208	0.148	0.147	0.428	0.018
30	0.0	0	-0.001	-0.014	0.126	0.126	-0.003	0.030	0.126	0.125	0.290	0.066
30	0.0	0.1	0.003	-0.006	0.126	0.126	-0.002	0.048	0.131	0.131	0.268	0.062
30	0.0	0.3	-0.003	0.004	0.125	0.125	-0.003	0.052	0.140	0.141	0.245	0.052
30	0.2	0.1	0.196	0.201	0.124	0.124	0.155	0.024	0.128	0.128	0.407	0.066
30	0.2	0.3	0.189	0.192	0.124	0.124	0.162	0.046	0.139	0.139	0.344	0.044
30	0.2	0.5	0.185	0.193	0.123	0.123	0.177	0.088	0.149	0.148	0.310	0.062
30	0.2	0.7	0.186	0.184	0.123	0.123	0.165	0.126	0.158	0.158	0.272	0.074
30	0.4	0.1	0.391	0.392	0.117	0.117	0.284	0.044	0.121	0.121	0.317	0.040
30	0.4	0.3	0.392	0.394	0.117	0.117	0.281	0.046	0.126	0.126	0.380	0.048
30	0.4	0.5	0.384	0.382	0.116	0.117	0.283	0.096	0.137	0.138	0.319	0.06
30	0.4	0.7	0.389	0.392	0.115	0.115	0.282	0.156	0.147	0.147	0.277	0.064
30	0.6	0.3	0.585	0.597	0.104	0.103	0.372	0.044	0.099	0.098	0.292	0.036
30	0.6	0.5	0.598	0.595	0.101	0.102	0.381	0.108	0.119	0.119	0.315	0.046
30	0.6	0.7	0.586	0.590	0.101	0.101	0.370	0.186	0.144	0.143	0.372	0.066
30	0.8	0.5	0.804	0.799	0.076	0.077	0.434	0.034	0.073	0.074	0.364	0.034
30	0.8	0.7	0.780	0.782	0.078	0.078	0.424	0.186	0.112	0.112	0.426	0.030
100	0.0	0.0	0.005	-0.001	0.070	0.070	-0.004	0.038	0.070	0.070	0.295	0.062
100	0.0	0.1	-0.004	-0.003	0.070	0.070	-0.001	0.020	0.074	0.074	0.276	0.046
100	0.0	0.3	-0.005	-0.004	0.070	0.070	0.001	0.062	0.080	0.080	0.236	0.062
100	0.2	0.1	0.195	0.199	0.069	0.069	0.165	0.028	0.071	0.071	0.412	0.050
100	0.2	0.3	0.201	0.204	0.069	0.069	0.167	0.048	0.077	0.077	0.347	0.054
100	0.2	0.5	0.201	0.196	0.069	0.069	0.162	0.102	0.083	0.083	0.298	0.064
100	0.2	0.7	0.193	0.202	0.069	0.069	0.172	0.120	0.089	0.089	0.265	0.060
100	0.4	0.1	0.397	0.397	0.065	0.065	0.277	0.042	0.067	0.067	0.314	0.046
100	0.4	0.3	0.397	0.394	0.065	0.065	0.279	0.036	0.070	0.070	0.387	0.040
100	0.4	0.5	0.397	0.404	0.064	0.064	0.281	0.110	0.076	0.076	0.317	0.072
100	0.4	0.7	0.398	0.403	0.064	0.064	0.294	0.120	0.082	0.082	0.285	0.042
100	0.6	0.3	0.599	0.603	0.056	0.056	0.374	0.062	0.054	0.054	0.309	0.066
100	0.6	0.5	0.599	0.598	0.056	0.056	0.374	0.116	0.066	0.066	0.315	0.052
100	0.6	0.7	0.601	0.595	0.056	0.056	0.373	0.178	0.079	0.080	0.373	0.070
100	0.8	0.5	0.800	0.799	0.042	0.042	0.440	0.066	0.040	0.040	0.371	0.064
100	0.8	0.7	0.796	0.798	0.042	0.042	0.442	0.160	0.060	0.060	0.442	0.038

Table 3: Simulation results obtained for a binary scale with uniform marginal distributions for $n_k = 4$ (Delta method)

Parameters					Delta method - classic				Delta method - multilevel			
K	κ	INT	κ_1	κ_2	$SE(\kappa_1)$	$SE(\kappa_2)$	$CORR$	α	$SE(\kappa_1)$	$SE(\kappa_2)$	$CORR$	α
20	0.0	0.0	-0.015	-0.012	0.110	0.110	-0.017	0.130	0.158	0.147	0.120	0.058
20	0.0	0.1	-0.011	-0.013	0.110	0.110	-0.029	0.040	0.130	0.129	0.368	0.050
20	0.0	0.3	-0.011	-0.019	0.109	0.109	-0.034	0.066	0.153	0.151	0.415	0.058
20	0.2	0.1	0.184	0.195	0.109	0.109	0.142	0.038	0.129	0.121	0.470	0.068
20	0.2	0.3	0.194	0.194	0.108	0.108	0.163	0.056	0.124	0.129	0.366	0.054
20	0.2	0.5	0.182	0.187	0.107	0.107	0.143	0.066	0.144	0.144	0.419	0.046
20	0.2	0.7	0.187	0.172	0.105	0.105	0.148	0.198	0.181	0.184	0.380	0.064
20	0.4	0.1	0.404	0.391	0.102	0.103	0.278	0.092	0.125	0.112	0.255	0.034
20	0.4	0.3	0.389	0.391	0.102	0.102	0.290	0.076	0.112	0.111	0.366	0.05
20	0.4	0.5	0.395	0.391	0.101	0.101	0.297	0.116	0.130	0.120	0.338	0.05
20	0.4	0.7	0.389	0.402	0.100	0.099	0.290	0.240	0.165	0.163	0.362	0.054
20	0.6	0.3	0.592	0.593	0.090	0.089	0.359	0.056	0.091	0.097	0.392	0.058
20	0.6	0.5	0.589	0.587	0.088	0.088	0.374	0.226	0.132	0.131	0.342	0.05
20	0.6	0.7	0.594	0.592	0.085	0.085	0.377	0.428	0.174	0.175	0.374	0.066
20	0.8	0.5	0.792	0.793	0.068	0.068	0.445	0.050	0.060	0.058	0.219	0.042
20	0.8	0.7	0.742	0.752	0.072	0.071	0.432	0.528	0.148	0.145	0.429	0.012
30	0.0	0.0	-0.003	-0.014	0.090	0.090	-0.016	0.124	0.129	0.120	0.126	0.052
30	0.0	0.1	0.00	0.004	0.090	0.090	-0.006	0.048	0.108	0.107	0.391	0.062
30	0.0	0.3	-0.001	-0.007	0.090	0.090	-0.017	0.056	0.127	0.125	0.425	0.05
30	0.2	0.1	0.196	0.196	0.089	0.089	0.157	0.022	0.107	0.100	0.489	0.046
30	0.2	0.3	0.188	0.184	0.089	0.089	0.160	0.050	0.102	0.105	0.359	0.054
30	0.2	0.5	0.182	0.185	0.088	0.088	0.154	0.074	0.120	0.119	0.445	0.052
30	0.2	0.7	0.181	0.185	0.087	0.087	0.163	0.200	0.150	0.152	0.386	0.086
30	0.4	0.1	0.393	0.390	0.084	0.084	0.284	0.086	0.103	0.092	0.280	0.052
30	0.4	0.3	0.398	0.401	0.083	0.083	0.283	0.072	0.090	0.091	0.361	0.064
30	0.4	0.5	0.386	0.394	0.083	0.083	0.292	0.130	0.106	0.099	0.319	0.066
30	0.4	0.7	0.392	0.387	0.082	0.082	0.29	0.228	0.134	0.135	0.363	0.056
30	0.6	0.3	0.597	0.596	0.073	0.073	0.362	0.06	0.074	0.080	0.412	0.056
30	0.6	0.5	0.594	0.593	0.072	0.072	0.382	0.204	0.108	0.107	0.348	0.048
30	0.6	0.7	0.598	0.593	0.071	0.071	0.366	0.386	0.144	0.145	0.364	0.054
30	0.8	0.5	0.796	0.795	0.055	0.055	0.431	0.050	0.048	0.047	0.201	0.040
30	0.8	0.7	0.785	0.779	0.054	0.055	0.423	0.500	0.111	0.112	0.422	0.032
100	0.0	0.0	-0.004	-0.003	0.050	0.050	-0.001	0.156	0.071	0.066	0.147	0.078
100	0.0	0.1	0.000	0.001	0.050	0.050	0.001	0.024	0.059	0.059	0.409	0.044
100	0.0	0.3	0.001	-0.002	0.050	0.050	-0.002	0.064	0.071	0.07	0.443	0.048
100	0.2	0.1	0.192	0.194	0.049	0.049	0.163	0.026	0.059	0.055	0.494	0.044
100	0.2	0.3	0.197	0.20	0.049	0.049	0.162	0.048	0.056	0.058	0.364	0.046
100	0.2	0.5	0.198	0.199	0.049	0.049	0.161	0.072	0.066	0.066	0.442	0.048
100	0.2	0.7	0.199	0.196	0.049	0.049	0.163	0.166	0.083	0.085	0.384	0.050
100	0.4	0.1	0.398	0.401	0.046	0.046	0.286	0.088	0.056	0.050	0.264	0.050
100	0.4	0.3	0.397	0.399	0.046	0.046	0.287	0.058	0.049	0.050	0.358	0.050
100	0.4	0.5	0.401	0.397	0.046	0.046	0.281	0.120	0.059	0.055	0.311	0.052
100	0.4	0.7	0.401	0.399	0.045	0.046	0.286	0.224	0.074	0.075	0.361	0.050
100	0.6	0.3	0.600	0.598	0.040	0.040	0.375	0.058	0.041	0.044	0.442	0.062
100	0.6	0.5	0.600	0.602	0.040	0.040	0.377	0.184	0.06	0.059	0.335	0.048
100	0.6	0.7	0.601	0.597	0.040	0.040	0.368	0.362	0.079	0.080	0.368	0.046
100	0.8	0.5	0.800	0.798	0.030	0.030	0.446	0.048	0.025	0.025	0.210	0.046
100	0.8	0.7	0.800	0.797	0.030	0.030	0.445	0.362	0.060	0.060	0.445	0.048

Table 4: Simulation results obtained for a binary scale with uniform marginal distributions for $n_k = 4$ (Bootstrap method)

Parameters			Delta method - classic						Bootstrap - multilevel			
K	κ	INT	κ_1	κ_2	$SE(\kappa_1)$	$SE(\kappa_2)$	$CORR$	α	$SE(\kappa_1)$	$SE(\kappa_2)$	$CORR$	α
20	0.0	0.0	-0.015	-0.012	0.110	0.110	-0.017	0.130	0.155	0.143	0.107	0.062
20	0.0	0.1	-0.011	-0.013	0.110	0.110	-0.029	0.040	0.127	0.126	0.359	0.060
20	0.0	0.3	-0.011	-0.019	0.109	0.109	-0.034	0.066	0.148	0.146	0.41	0.060
20	0.2	0.1	0.184	0.195	0.109	0.109	0.142	0.038	0.127	0.119	0.465	0.070
20	0.2	0.3	0.194	0.194	0.108	0.108	0.163	0.056	0.122	0.126	0.365	0.054
20	0.2	0.5	0.182	0.187	0.107	0.107	0.143	0.066	0.141	0.141	0.416	0.044
20	0.2	0.7	0.187	0.172	0.105	0.105	0.148	0.198	0.175	0.178	0.378	0.060
20	0.4	0.1	0.404	0.391	0.102	0.103	0.278	0.092	0.124	0.112	0.259	0.050
20	0.4	0.3	0.389	0.391	0.102	0.102	0.29	0.076	0.111	0.11	0.369	0.056
20	0.4	0.5	0.395	0.391	0.101	0.101	0.297	0.116	0.128	0.119	0.349	0.052
20	0.4	0.7	0.389	0.402	0.1	0.099	0.29	0.240	0.161	0.159	0.365	0.058
20	0.6	0.3	0.592	0.593	0.09	0.089	0.359	0.056	0.090	0.096	0.389	0.064
20	0.6	0.5	0.589	0.587	0.088	0.088	0.374	0.226	0.129	0.129	0.352	0.056
20	0.6	0.7	0.594	0.592	0.085	0.085	0.377	0.428	0.17	0.171	0.38	0.066
20	0.8	0.5	0.792	0.793	0.068	0.068	0.445	0.05	0.063	0.062	0.253	0.040
20	0.8	0.7	0.742	0.752	0.072	0.071	0.432	0.528	0.147	0.143	0.434	0.012
30	0.0	0.0	-0.003	-0.014	0.090	0.090	-0.016	0.124	0.127	0.118	0.118	0.058
30	0.0	0.1	0	0.004	0.090	0.090	-0.006	0.048	0.107	0.105	0.384	0.068
30	0.0	0.3	-0.001	-0.007	0.090	0.090	-0.017	0.056	0.124	0.123	0.422	0.050
30	0.2	0.1	0.196	0.196	0.089	0.089	0.157	0.022	0.105	0.099	0.485	0.048
30	0.2	0.3	0.188	0.184	0.089	0.089	0.16	0.05	0.1	0.103	0.357	0.056
30	0.2	0.5	0.182	0.185	0.088	0.088	0.154	0.074	0.118	0.117	0.443	0.050
30	0.2	0.7	0.181	0.185	0.087	0.087	0.163	0.200	0.147	0.149	0.385	0.088
30	0.4	0.1	0.393	0.39	0.084	0.084	0.284	0.086	0.102	0.092	0.283	0.060
30	0.4	0.3	0.398	0.401	0.083	0.083	0.283	0.072	0.089	0.09	0.362	0.066
30	0.4	0.5	0.386	0.394	0.083	0.083	0.292	0.130	0.105	0.098	0.326	0.068
30	0.4	0.7	0.392	0.387	0.082	0.082	0.29	0.228	0.132	0.133	0.365	0.060
30	0.6	0.3	0.597	0.596	0.073	0.073	0.362	0.06	0.074	0.08	0.409	0.062
30	0.6	0.5	0.594	0.593	0.072	0.072	0.382	0.204	0.107	0.106	0.354	0.056
30	0.6	0.7	0.598	0.593	0.071	0.071	0.366	0.386	0.142	0.143	0.369	0.054
30	0.8	0.5	0.796	0.795	0.055	0.055	0.431	0.05	0.05	0.049	0.225	0.050
30	0.8	0.7	0.785	0.779	0.054	0.055	0.423	0.500	0.11	0.112	0.424	0.032
100	0.0	0.0	-0.004	-0.003	0.050	0.050	-0.001	0.156	0.071	0.066	0.145	0.080
100	0.0	0.1	0.000	0.001	0.050	0.050	0.001	0.024	0.059	0.059	0.407	0.044
100	0.0	0.3	0.001	-0.002	0.050	0.050	-0.002	0.064	0.071	0.069	0.442	0.048
100	0.2	0.1	0.192	0.194	0.049	0.049	0.163	0.026	0.059	0.055	0.493	0.044
100	0.2	0.3	0.197	0.2	0.049	0.049	0.162	0.048	0.056	0.058	0.364	0.046
100	0.2	0.5	0.198	0.199	0.049	0.049	0.161	0.072	0.066	0.065	0.441	0.050
100	0.2	0.7	0.199	0.196	0.049	0.049	0.163	0.166	0.082	0.084	0.384	0.052
100	0.4	0.1	0.398	0.401	0.046	0.046	0.286	0.088	0.056	0.05	0.266	0.052
100	0.4	0.3	0.397	0.399	0.046	0.046	0.287	0.058	0.049	0.050	0.359	0.052
100	0.4	0.5	0.401	0.397	0.046	0.046	0.281	0.120	0.059	0.055	0.313	0.052
100	0.4	0.7	0.401	0.399	0.045	0.046	0.286	0.224	0.074	0.074	0.361	0.052
100	0.6	0.3	0.600	0.598	0.04	0.040	0.375	0.058	0.041	0.043	0.442	0.060
100	0.6	0.5	0.600	0.602	0.04	0.040	0.377	0.184	0.06	0.059	0.338	0.048
100	0.6	0.7	0.601	0.597	0.04	0.040	0.368	0.362	0.079	0.079	0.37	0.046
100	0.8	0.5	0.800	0.798	0.03	0.030	0.446	0.048	0.026	0.025	0.218	0.050
100	0.8	0.7	0.800	0.797	0.03	0.030	0.445	0.362	0.059	0.06	0.445	0.046

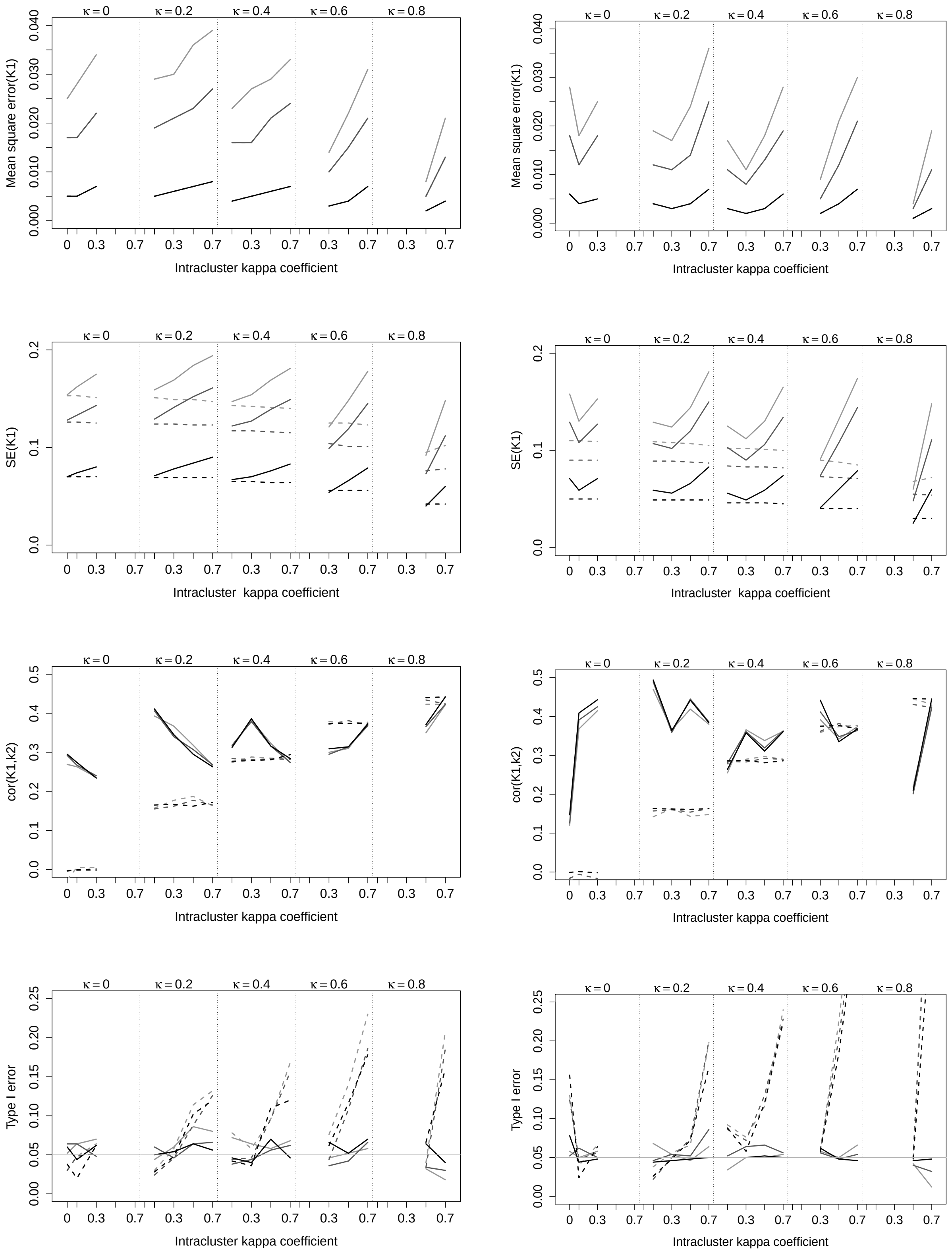

Figure 1: Mean squared error and mean standard error of κ_1, mean correlation between κ_1 and κ_2 and type I error for the comparison of the two dependent multilevel kappa coefficients obtained on a binary scale when the observers marginal probability distribution is uniform and the cluster size is equal to $n_k = 2$ (left) and $n_k = 4$ (right). The results obtained by the delta method ignoring the hierarchical structure (dashed lines) and by the hierarchical delta method (plain line) are reported for $K = 100$ (black), $K = 30$ (middle gray) and $K = 20$ (light gray) clusters

Table 5: Delta method. Simulation results obtained for a binary scale with (0.7,0.3) marginal distributions when $n_k = 2$

Parameters					Delta method - classic				Delta - multilevel			
K	κ	INT	κ_1	κ_2	$SE(\kappa_1)$	$SE(\kappa_2)$	$CORR$	α	$SE(\kappa_1)$	$SE(\kappa_2)$	$CORR$	α
20	0.0	0.0	-0.004	-0.010	0.143	0.143	0.271	0.026	0.173	0.173	0.555	0.040
20	0.0	0.1	0.004	-0.017	0.143	0.140	0.322	0.024	0.176	0.174	0.581	0.030
20	0.0	0.3	-0.011	-0.014	0.140	0.140	0.428	0.054	0.181	0.181	0.638	0.044
20	0.2	0.1	0.181	0.170	0.153	0.151	0.284	0.050	0.185	0.184	0.548	0.050
20	0.2	0.3	0.193	0.187	0.151	0.150	0.345	0.076	0.190	0.189	0.561	0.066
20	0.2	0.5	0.18	0.190	0.149	0.148	0.425	0.086	0.197	0.196	0.579	0.052
20	0.2	0.7	0.186	0.183	0.147	0.148	0.501	0.096	0.203	0.204	0.593	0.054
20	0.4	0.1	0.391	0.374	0.151	0.152	0.171	0.054	0.177	0.180	0.321	0.032
20	0.4	0.3	0.382	0.385	0.151	0.151	0.272	0.060	0.183	0.183	0.467	0.040
20	0.4	0.5	0.366	0.357	0.152	0.151	0.341	0.104	0.193	0.192	0.473	0.054
20	0.4	0.7	0.374	0.375	0.147	0.148	0.403	0.134	0.197	0.198	0.481	0.048
20	0.6	0.3	0.583	0.592	0.137	0.135	0.196	0.076	0.152	0.150	0.286	0.042
20	0.6	0.5	0.584	0.596	0.135	0.134	0.317	0.104	0.168	0.165	0.374	0.036
20	0.6	0.7	0.560	0.573	0.136	0.135	0.556	0.236	0.194	0.193	0.542	0.052
20	0.8	0.5	0.780	0.787	0.107	0.105	0.342	0.042	0.113	0.111	0.364	0.018
20	0.8	0.7	0.715	0.718	0.118	0.118	0.534	0.184	0.169	0.169	0.525	0.008
30	0.0	0.0	-0.014	-0.006	0.120	0.121	0.315	0.030	0.147	0.148	0.598	0.036
30	0.0	0.1	0.008	-0.005	0.122	0.122	0.378	0.034	0.151	0.152	0.639	0.052
30	0.0	0.3	-0.001	0.003	0.120	0.120	0.465	0.064	0.156	0.156	0.667	0.072
30	0.2	0.1	0.195	0.191	0.128	0.128	0.305	0.040	0.154	0.156	0.577	0.064
30	0.2	0.3	0.178	0.194	0.127	0.128	0.367	0.054	0.161	0.161	0.583	0.048
30	0.2	0.5	0.184	0.176	0.127	0.127	0.456	0.080	0.167	0.167	0.614	0.060
30	0.2	0.7	0.181	0.185	0.126	0.125	0.523	0.104	0.172	0.171	0.627	0.050
30	0.4	0.1	0.393	0.391	0.126	0.126	0.230	0.084	0.148	0.148	0.389	0.062
30	0.4	0.3	0.392	0.392	0.125	0.125	0.300	0.070	0.152	0.152	0.501	0.056
30	0.4	0.5	0.382	0.387	0.125	0.125	0.367	0.072	0.160	0.159	0.510	0.040
30	0.4	0.7	0.394	0.394	0.123	0.124	0.409	0.098	0.165	0.165	0.490	0.042
30	0.6	0.3	0.585	0.602	0.113	0.111	0.222	0.074	0.124	0.122	0.322	0.052
30	0.6	0.5	0.586	0.589	0.111	0.111	0.355	0.128	0.138	0.137	0.417	0.062
30	0.6	0.7	0.582	0.584	0.111	0.111	0.556	0.228	0.156	0.157	0.543	0.066
30	0.8	0.5	0.796	0.804	0.085	0.083	0.334	0.046	0.088	0.086	0.358	0.026
30	0.8	0.7	0.777	0.767	0.087	0.088	0.528	0.224	0.123	0.126	0.522	0.026
100	0.0	0.0	-0.010	-0.011	0.069	0.069	0.372	0.036	0.085	0.085	0.661	0.060
100	0.0	0.1	-0.008	-0.004	0.069	0.069	0.413	0.034	0.086	0.087	0.674	0.062
100	0.0	0.3	-0.008	-0.010	0.069	0.069	0.514	0.060	0.090	0.090	0.711	0.066
100	0.2	0.1	0.206	0.199	0.072	0.072	0.329	0.030	0.087	0.087	0.607	0.042
100	0.2	0.3	0.199	0.200	0.072	0.072	0.399	0.050	0.091	0.091	0.616	0.054
100	0.2	0.5	0.196	0.194	0.072	0.072	0.476	0.088	0.094	0.094	0.633	0.062
100	0.2	0.7	0.203	0.202	0.072	0.072	0.553	0.078	0.098	0.098	0.643	0.034
100	0.4	0.1	0.398	0.393	0.070	0.070	0.231	0.054	0.082	0.082	0.395	0.040
100	0.4	0.3	0.399	0.396	0.070	0.070	0.310	0.048	0.084	0.084	0.519	0.042
100	0.4	0.5	0.396	0.392	0.070	0.070	0.368	0.088	0.088	0.089	0.514	0.060
100	0.4	0.7	0.396	0.398	0.070	0.070	0.428	0.096	0.092	0.092	0.512	0.048
100	0.6	0.3	0.593	0.594	0.062	0.062	0.231	0.056	0.068	0.068	0.345	0.044
100	0.6	0.5	0.591	0.591	0.062	0.062	0.366	0.096	0.077	0.077	0.436	0.048
100	0.6	0.7	0.592	0.591	0.062	0.062	0.568	0.178	0.087	0.087	0.556	0.038
100	0.8	0.5	0.795	0.794	0.047	0.047	0.348	0.036	0.048	0.049	0.378	0.030
100	0.8	0.7	0.798	0.801	0.046	0.046	0.526	0.184	0.065	0.065	0.521	0.034

Table 6: Bootstrap method. Simulation results obtained for a binary scale with (0.7,0.3) marginal distributions when $n_k = 2$

Parameters					Delta method - classic				Bootstrap - multilevel			
K	κ	INT	κ_1	κ_2	$SE(\kappa_1)$	$SE(\kappa_2)$	$CORR$	α	$SE(\kappa_1)$	$SE(\kappa_2)$	$CORR$	α
20	0.0	0.0	-0.004	-0.010	0.143	0.143	0.271	0.026	0.167	0.167	0.537	0.038
20	0.0	0.1	0.004	-0.017	0.143	0.140	0.322	0.024	0.17	0.168	0.565	0.032
20	0.0	0.3	-0.011	-0.014	0.140	0.140	0.428	0.054	0.174	0.174	0.626	0.036
20	0.2	0.1	0.181	0.170	0.153	0.151	0.284	0.050	0.181	0.179	0.531	0.056
20	0.2	0.3	0.193	0.187	0.151	0.150	0.345	0.076	0.186	0.185	0.549	0.066
20	0.2	0.5	0.18	0.190	0.149	0.148	0.425	0.086	0.192	0.191	0.574	0.044
20	0.2	0.7	0.186	0.183	0.147	0.148	0.501	0.096	0.198	0.200	0.600	0.048
20	0.4	0.1	0.391	0.374	0.151	0.152	0.171	0.054	0.177	0.179	0.301	0.034
20	0.4	0.3	0.382	0.385	0.151	0.151	0.272	0.060	0.182	0.183	0.450	0.044
20	0.4	0.5	0.366	0.357	0.152	0.151	0.341	0.104	0.192	0.191	0.465	0.046
20	0.4	0.7	0.374	0.375	0.147	0.148	0.403	0.134	0.195	0.196	0.483	0.050
20	0.6	0.3	0.583	0.592	0.137	0.135	0.196	0.076	0.157	0.155	0.263	0.044
20	0.6	0.5	0.584	0.596	0.135	0.134	0.317	0.104	0.170	0.169	0.369	0.040
20	0.6	0.7	0.560	0.573	0.136	0.135	0.556	0.236	0.195	0.194	0.561	0.040
20	0.8	0.5	0.780	0.787	0.107	0.105	0.342	0.042	0.125	0.123	0.351	0.018
20	0.8	0.7	0.715	0.718	0.118	0.118	0.534	0.184	0.173	0.173	0.549	0.006
30	0.0	0.0	-0.014	-0.006	0.120	0.121	0.315	0.030	0.143	0.143	0.585	0.036
30	0.0	0.1	0.008	-0.005	0.122	0.122	0.378	0.034	0.147	0.147	0.629	0.046
30	0.0	0.3	-0.001	0.003	0.120	0.120	0.465	0.064	0.151	0.152	0.659	0.068
30	0.2	0.1	0.195	0.191	0.128	0.128	0.305	0.040	0.152	0.153	0.565	0.064
30	0.2	0.3	0.178	0.194	0.127	0.128	0.367	0.054	0.157	0.158	0.575	0.046
30	0.2	0.5	0.184	0.176	0.127	0.127	0.456	0.080	0.164	0.163	0.611	0.062
30	0.2	0.7	0.181	0.185	0.126	0.125	0.523	0.104	0.169	0.168	0.630	0.048
30	0.4	0.1	0.393	0.391	0.126	0.126	0.230	0.084	0.148	0.148	0.375	0.066
30	0.4	0.3	0.392	0.392	0.125	0.125	0.300	0.070	0.152	0.151	0.492	0.056
30	0.4	0.5	0.382	0.387	0.125	0.125	0.367	0.072	0.159	0.159	0.506	0.042
30	0.4	0.7	0.394	0.394	0.123	0.124	0.409	0.098	0.164	0.164	0.495	0.044
30	0.6	0.3	0.585	0.602	0.113	0.111	0.222	0.074	0.127	0.124	0.308	0.054
30	0.6	0.5	0.586	0.589	0.111	0.111	0.355	0.128	0.139	0.139	0.415	0.064
30	0.6	0.7	0.582	0.584	0.111	0.111	0.556	0.228	0.156	0.157	0.556	0.066
30	0.8	0.5	0.796	0.804	0.085	0.083	0.334	0.046	0.094	0.092	0.353	0.026
30	0.8	0.7	0.777	0.767	0.087	0.088	0.528	0.224	0.126	0.128	0.535	0.026
100	0.0	0.0	-0.010	-0.011	0.069	0.069	0.372	0.036	0.084	0.084	0.657	0.058
100	0.0	0.1	-0.008	-0.004	0.069	0.069	0.413	0.034	0.086	0.086	0.671	0.058
100	0.0	0.3	-0.008	-0.010	0.069	0.069	0.514	0.060	0.089	0.089	0.710	0.064
100	0.2	0.1	0.206	0.199	0.072	0.072	0.329	0.030	0.087	0.087	0.603	0.042
100	0.2	0.3	0.199	0.200	0.072	0.072	0.399	0.050	0.090	0.091	0.615	0.056
100	0.2	0.5	0.196	0.194	0.072	0.072	0.476	0.088	0.094	0.094	0.633	0.06
100	0.2	0.7	0.203	0.202	0.072	0.072	0.553	0.078	0.098	0.098	0.647	0.034
100	0.4	0.1	0.398	0.393	0.070	0.070	0.231	0.054	0.082	0.082	0.390	0.046
100	0.4	0.3	0.399	0.396	0.070	0.070	0.310	0.048	0.084	0.084	0.517	0.044
100	0.4	0.5	0.396	0.392	0.070	0.070	0.368	0.088	0.088	0.088	0.515	0.060
100	0.4	0.7	0.396	0.398	0.070	0.070	0.428	0.096	0.092	0.092	0.514	0.046
100	0.6	0.3	0.593	0.594	0.062	0.062	0.231	0.056	0.068	0.068	0.340	0.044
100	0.6	0.5	0.591	0.591	0.062	0.062	0.366	0.096	0.077	0.077	0.436	0.044
100	0.6	0.7	0.592	0.591	0.062	0.062	0.568	0.178	0.087	0.087	0.561	0.04
100	0.8	0.5	0.795	0.794	0.047	0.047	0.348	0.036	0.049	0.049	0.378	0.030
100	0.8	0.7	0.798	0.801	0.046	0.046	0.526	0.184	0.066	0.065	0.525	0.036

Table 7: Delta method. Simulation results obtained for a binary scale with (0.7,0.3) marginal distributions when $n_k = 4$

Parameters					Delta method - classic				Delta - multilevel			
K	κ	INT	κ_1	κ_2	$SE(\kappa_1)$	$SE(\kappa_2)$	$CORR$	α	$SE(\kappa_1)$	$SE(\kappa_2)$	$CORR$	α
20	0.0	0.0	0.003	-0.008	0.111	0.110	-0.138	0.058	0.113	0.133	0.161	0.068
20	0.0	0.1	-0.012	-0.010	0.109	0.109	-0.149	0.008	0.106	0.101	0.251	0.052
20	0.0	0.3	-0.003	-0.008	0.108	0.109	-0.180	0.048	0.128	0.125	0.300	0.078
20	0.2	0.1	0.200	0.186	0.115	0.116	0.068	0.066	0.125	0.102	-0.004	0.066
20	0.2	0.3	0.180	0.176	0.109	0.110	0.359	0.028	0.138	0.135	0.613	0.056
20	0.2	0.5	0.200	0.199	0.112	0.112	-0.008	0.060	0.134	0.146	0.308	0.064
20	0.2	0.7	0.182	0.190	0.108	0.110	-0.162	0.224	0.179	0.164	-0.051	0.118
20	0.4	0.1	0.384	0.392	0.111	0.110	0.219	0.118	0.126	0.148	0.116	0.054
20	0.4	0.3	0.379	0.379	0.109	0.109	0.351	0.056	0.134	0.139	0.523	0.042
20	0.4	0.5	0.378	0.380	0.107	0.108	0.319	0.108	0.149	0.152	0.483	0.048
20	0.4	0.7	0.396	0.389	0.106	0.107	0.115	0.238	0.174	0.162	0.090	0.106
20	0.6	0.3	0.582	0.579	0.099	0.099	0.203	0.074	0.115	0.115	0.367	0.042
20	0.6	0.5	0.595	0.602	0.096	0.096	0.337	0.170	0.131	0.130	0.377	0.078
20	0.6	0.7	0.579	0.568	0.094	0.096	0.181	0.456	0.188	0.191	0.157	0.078
20	0.8	0.5	0.792	0.791	0.074	0.074	0.404	0.054	0.082	0.082	0.516	0.04
20	0.8	0.7	0.732	0.733	0.082	0.082	0.536	0.548	0.165	0.165	0.524	0.006
30	0.0	0.0	-0.001	-0.012	0.091	0.090	-0.146	0.052	0.093	0.110	0.138	0.070
30	0.0	0.1	-0.007	-0.006	0.090	0.090	-0.115	0.012	0.091	0.086	0.297	0.072
30	0.0	0.3	-0.012	-0.003	0.089	0.090	-0.192	0.038	0.106	0.102	0.279	0.056
30	0.2	0.1	0.191	0.197	0.094	0.094	0.075	0.068	0.104	0.083	-0.001	0.072
30	0.2	0.3	0.177	0.179	0.092	0.092	0.381	0.024	0.120	0.116	0.681	0.048
30	0.2	0.5	0.200	0.188	0.093	0.093	-0.024	0.050	0.111	0.121	0.302	0.048
30	0.2	0.7	0.181	0.198	0.091	0.092	-0.156	0.226	0.149	0.138	-0.048	0.078
30	0.4	0.1	0.393	0.397	0.091	0.091	0.222	0.140	0.104	0.120	0.098	0.05
30	0.4	0.3	0.386	0.394	0.090	0.090	0.348	0.066	0.111	0.115	0.535	0.050
30	0.4	0.5	0.384	0.383	0.089	0.089	0.332	0.114	0.126	0.126	0.505	0.038
30	0.4	0.7	0.385	0.400	0.088	0.088	0.102	0.252	0.146	0.134	0.098	0.062
30	0.6	0.3	0.590	0.593	0.080	0.080	0.211	0.036	0.092	0.092	0.371	0.016
30	0.6	0.5	0.592	0.598	0.079	0.079	0.320	0.152	0.111	0.110	0.380	0.056
30	0.6	0.7	0.600	0.600	0.078	0.078	0.202	0.428	0.154	0.154	0.175	0.072
30	0.8	0.5	0.797	0.796	0.060	0.061	0.420	0.056	0.067	0.067	0.527	0.060
30	0.8	0.7	0.773	0.771	0.062	0.062	0.526	0.504	0.124	0.125	0.516	0.026
100	0.0	0.0	-0.002	-0.005	0.050	0.050	-0.141	0.054	0.051	0.061	0.123	0.052
100	0.0	0.1	-0.001	-0.003	0.050	0.050	-0.114	0.008	0.051	0.049	0.322	0.066
100	0.0	0.3	-0.005	0.000	0.050	0.050	-0.191	0.042	0.059	0.057	0.271	0.062
100	0.2	0.1	0.201	0.197	0.052	0.052	0.070	0.062	0.057	0.046	-0.018	0.048
100	0.2	0.3	0.200	0.199	0.051	0.051	0.395	0.032	0.067	0.065	0.704	0.054
100	0.2	0.5	0.200	0.201	0.051	0.051	-0.009	0.036	0.063	0.068	0.375	0.036
100	0.2	0.7	0.197	0.204	0.051	0.051	-0.172	0.194	0.086	0.077	-0.093	0.046
100	0.4	0.1	0.399	0.399	0.050	0.050	0.223	0.142	0.058	0.066	0.130	0.048
100	0.4	0.3	0.396	0.394	0.050	0.050	0.366	0.064	0.061	0.064	0.559	0.046
100	0.4	0.5	0.401	0.400	0.049	0.049	0.358	0.096	0.069	0.070	0.534	0.040
100	0.4	0.7	0.393	0.391	0.049	0.049	0.089	0.228	0.082	0.075	0.081	0.056
100	0.6	0.3	0.594	0.595	0.044	0.044	0.224	0.046	0.051	0.051	0.410	0.046
100	0.6	0.5	0.596	0.600	0.044	0.044	0.334	0.178	0.063	0.061	0.418	0.052
100	0.6	0.7	0.597	0.598	0.043	0.043	0.171	0.378	0.086	0.085	0.141	0.072
100	0.8	0.5	0.796	0.795	0.033	0.033	0.427	0.060	0.037	0.037	0.550	0.076
100	0.8	0.7	0.794	0.797	0.033	0.033	0.534	0.348	0.066	0.065	0.527	0.038

Table 8: Bootstrap method. Simulation results obtained for a binary scale with (0.7,0.3) marginal distributions when $n_k = 4$

Parameters					Delta method - classic				Bootstrap - multilevel			
K	κ	INT	κ_1	κ_2	$SE(\kappa_1)$	$SE(\kappa_2)$	$CORR$	α	$SE(\kappa_1)$	$SE(\kappa_2)$	$CORR$	α
20	0.0	0.0	0.003	-0.008	0.111	0.11	-0.138	0.058	0.111	0.131	0.158	0.072
20	0.0	0.1	-0.012	-0.010	0.109	0.109	-0.149	0.008	0.104	0.099	0.261	0.054
20	0.0	0.3	-0.003	-0.008	0.108	0.109	-0.180	0.048	0.125	0.122	0.296	0.078
20	0.2	0.1	0.200	0.186	0.115	0.116	0.068	0.066	0.123	0.101	-0.001	0.068
20	0.2	0.3	0.180	0.176	0.109	0.110	0.359	0.028	0.137	0.133	0.630	0.052
20	0.2	0.5	0.200	0.199	0.112	0.112	-0.008	0.060	0.132	0.144	0.284	0.060
20	0.2	0.7	0.182	0.190	0.108	0.110	-0.162	0.224	0.175	0.161	-0.050	0.112
20	0.4	0.1	0.384	0.392	0.111	0.110	0.219	0.118	0.125	0.147	0.110	0.054
20	0.4	0.3	0.379	0.379	0.109	0.109	0.351	0.056	0.136	0.140	0.532	0.048
20	0.4	0.5	0.378	0.380	0.107	0.108	0.319	0.108	0.148	0.152	0.488	0.048
20	0.4	0.7	0.396	0.389	0.106	0.107	0.115	0.238	0.172	0.161	0.083	0.106
20	0.6	0.3	0.582	0.579	0.099	0.099	0.203	0.074	0.119	0.119	0.350	0.044
20	0.6	0.5	0.595	0.602	0.096	0.096	0.337	0.170	0.130	0.129	0.374	0.080
20	0.6	0.7	0.579	0.568	0.094	0.096	0.181	0.456	0.188	0.190	0.165	0.082
20	0.8	0.5	0.792	0.791	0.074	0.074	0.404	0.054	0.086	0.085	0.500	0.044
20	0.8	0.7	0.732	0.733	0.082	0.082	0.536	0.548	0.167	0.167	0.537	0.006
30	0.0	0.0	-0.001	-0.012	0.091	0.090	-0.146	0.052	0.092	0.109	0.136	0.074
30	0.0	0.1	-0.007	-0.006	0.090	0.090	-0.115	0.012	0.089	0.085	0.302	0.072
30	0.0	0.3	-0.012	-0.003	0.089	0.090	-0.192	0.038	0.104	0.100	0.277	0.056
30	0.2	0.1	0.191	0.197	0.094	0.094	0.075	0.068	0.103	0.082	0.001	0.076
30	0.2	0.3	0.177	0.179	0.092	0.092	0.381	0.024	0.119	0.115	0.691	0.046
30	0.2	0.5	0.200	0.188	0.093	0.093	-0.024	0.050	0.109	0.119	0.286	0.050
30	0.2	0.7	0.181	0.198	0.091	0.092	-0.156	0.226	0.146	0.136	-0.047	0.076
30	0.4	0.1	0.393	0.397	0.091	0.091	0.222	0.140	0.104	0.119	0.094	0.060
30	0.4	0.3	0.386	0.394	0.090	0.090	0.348	0.066	0.112	0.116	0.545	0.050
30	0.4	0.5	0.384	0.383	0.089	0.089	0.332	0.114	0.125	0.126	0.511	0.040
30	0.4	0.7	0.385	0.400	0.088	0.088	0.102	0.252	0.144	0.133	0.092	0.058
30	0.6	0.3	0.590	0.593	0.080	0.080	0.211	0.036	0.095	0.094	0.363	0.016
30	0.6	0.5	0.592	0.598	0.079	0.079	0.320	0.152	0.110	0.109	0.380	0.054
30	0.6	0.7	0.600	0.600	0.078	0.078	0.202	0.428	0.153	0.153	0.179	0.068
30	0.8	0.5	0.797	0.796	0.060	0.061	0.420	0.056	0.069	0.068	0.520	0.062
30	0.8	0.7	0.773	0.771	0.062	0.062	0.526	0.504	0.125	0.126	0.526	0.026
100	0.0	0.0	-0.002	-0.005	0.05	0.050	-0.141	0.054	0.051	0.061	0.122	0.050
100	0.0	0.1	-0.001	-0.003	0.05	0.050	-0.114	0.008	0.050	0.049	0.323	0.066
100	0.0	0.3	-0.005	0.000	0.05	0.050	-0.191	0.042	0.059	0.057	0.270	0.062
100	0.2	0.1	0.201	0.197	0.052	0.052	0.070	0.062	0.057	0.046	-0.018	0.046
100	0.2	0.3	0.200	0.199	0.051	0.051	0.395	0.032	0.067	0.065	0.708	0.056
100	0.2	0.5	0.200	0.201	0.051	0.051	-0.009	0.036	0.063	0.068	0.370	0.038
100	0.2	0.7	0.197	0.204	0.051	0.051	-0.172	0.194	0.085	0.077	-0.093	0.046
100	0.4	0.1	0.399	0.399	0.050	0.050	0.223	0.142	0.058	0.066	0.128	0.05
100	0.4	0.3	0.396	0.394	0.050	0.050	0.366	0.064	0.062	0.064	0.563	0.046
100	0.4	0.5	0.401	0.400	0.049	0.049	0.358	0.096	0.069	0.070	0.537	0.04
100	0.4	0.7	0.393	0.391	0.049	0.049	0.089	0.228	0.082	0.075	0.08	0.058
100	0.6	0.3	0.594	0.595	0.044	0.044	0.224	0.046	0.051	0.051	0.409	0.048
100	0.6	0.5	0.596	0.600	0.044	0.044	0.334	0.178	0.063	0.061	0.417	0.054
100	0.6	0.7	0.597	0.598	0.043	0.043	0.171	0.378	0.085	0.085	0.143	0.072
100	0.8	0.5	0.796	0.795	0.033	0.033	0.427	0.06	0.038	0.038	0.548	0.074
100	0.8	0.7	0.794	0.797	0.033	0.033	0.534	0.348	0.066	0.065	0.529	0.038

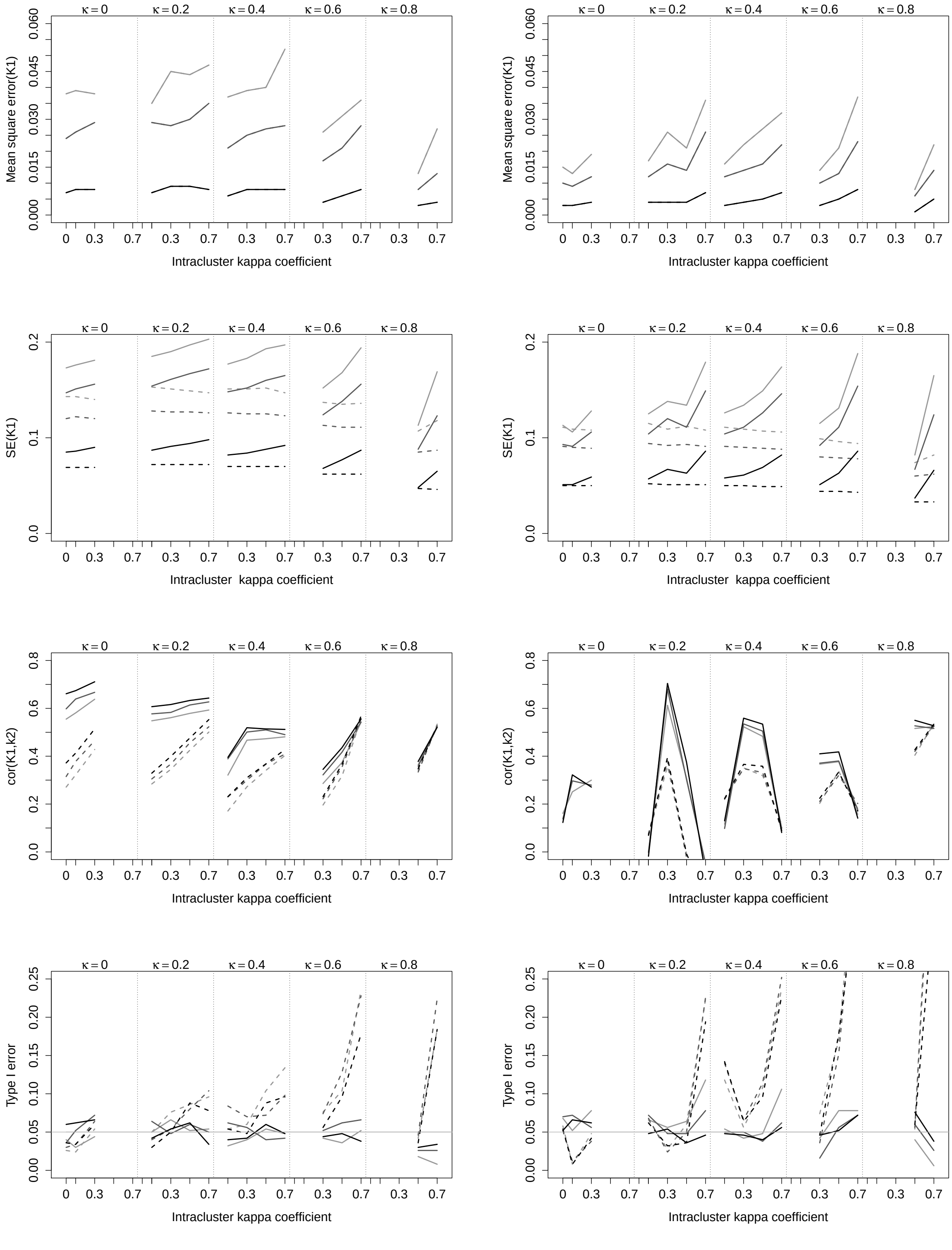

Figure 2: Mean squared error and mean standard error of κ_1, mean correlation between κ_1 and κ_2 and type I error for the comparison of the two dependent multilevel kappa coefficients obtained on a binary scale when the observers marginal probability distribution is (0.7,0.3) and the cluster size is equal to $n_k = 2$ (left) and $n_k = 4$ (right). The results obtained by the delta method ignoring the hierarchical structure (dashed lines) and by the hierarchical delta method (plain line) are reported for $K = 100$ (black), $K = 30$ (middle gray) and $K = 20$ (light gray) clusters

Table 9: Delta method. Simulation results obtained for a 3-ordinal scale with uniform marginal distributions when $n_k = 2$

Parameters					Delta method - classic				Delta - multilevel			
K	κ	INT	κ_1	κ_2	$SE(\kappa_1)$	$SE(\kappa_2)$	$CORR$	α	$SE(\kappa_1)$	$SE(\kappa_2)$	$CORR$	α
20	0.0	0.0	-0.003	-0.005	0.120	0.120	-0.008	0.048	0.141	0.125	0.140	0.054
20	0.0	0.1	-0.003	-0.009	0.120	0.118	-0.087	0.056	0.129	0.129	0.094	0.052
20	0.0	0.3	-0.002	-0.019	0.118	0.117	-0.058	0.100	0.146	0.138	0.050	0.074
20	0.2	0.1	0.191	0.198	0.127	0.127	0.107	0.038	0.123	0.120	0.149	0.044
20	0.2	0.3	0.196	0.195	0.126	0.126	0.143	0.032	0.130	0.125	0.282	0.040
20	0.2	0.5	0.200	0.204	0.123	0.123	0.276	0.110	0.153	0.152	0.388	0.072
20	0.2	0.7	0.201	0.174	0.122	0.123	0.074	0.130	0.158	0.162	0.116	0.048
20	0.4	0.1	0.393	0.380	0.123	0.122	0.354	0.098	0.144	0.147	0.406	0.058
20	0.4	0.3	0.398	0.393	0.122	0.122	0.372	0.072	0.139	0.134	0.464	0.072
20	0.4	0.5	0.398	0.393	0.122	0.123	0.397	0.056	0.136	0.122	0.390	0.042
20	0.4	0.7	0.393	0.384	0.119	0.121	0.391	0.108	0.153	0.153	0.511	0.058
20	0.6	0.3	0.586	0.586	0.111	0.111	0.383	0.060	0.119	0.118	0.397	0.042
20	0.6	0.5	0.593	0.594	0.109	0.109	0.521	0.070	0.134	0.135	0.650	0.044
20	0.6	0.7	0.588	0.589	0.107	0.107	0.875	0.236	0.154	0.154	0.874	0.070
20	0.8	0.5	0.800	0.800	0.082	0.082	0.441	0.032	0.092	0.093	0.518	0.016
20	0.8	0.7	0.768	0.778	0.086	0.084	0.521	0.216	0.124	0.122	0.519	0.022
30	0.0	0.0	-0.001	-0.006	0.099	0.099	-0.018	0.070	0.116	0.103	0.134	0.066
30	0.0	0.1	0.000	-0.004	0.099	0.099	-0.082	0.076	0.106	0.108	0.122	0.084
30	0.0	0.3	-0.013	-0.012	0.098	0.098	-0.047	0.086	0.122	0.114	0.060	0.056
30	0.2	0.1	0.189	0.190	0.104	0.104	0.109	0.032	0.102	0.099	0.164	0.048
30	0.2	0.3	0.188	0.201	0.104	0.103	0.131	0.050	0.109	0.102	0.286	0.066
30	0.2	0.5	0.185	0.189	0.102	0.102	0.285	0.092	0.127	0.126	0.404	0.064
30	0.2	0.7	0.187	0.190	0.102	0.102	0.071	0.116	0.131	0.134	0.103	0.062
30	0.4	0.1	0.405	0.398	0.101	0.101	0.338	0.104	0.118	0.122	0.378	0.05
30	0.4	0.3	0.392	0.388	0.101	0.101	0.377	0.044	0.115	0.111	0.474	0.052
30	0.4	0.5	0.387	0.389	0.101	0.101	0.388	0.092	0.112	0.101	0.364	0.064
30	0.4	0.7	0.393	0.396	0.100	0.100	0.407	0.100	0.128	0.126	0.522	0.052
30	0.6	0.3	0.587	0.593	0.092	0.091	0.390	0.058	0.099	0.096	0.410	0.044
30	0.6	0.5	0.594	0.595	0.090	0.090	0.519	0.064	0.110	0.112	0.645	0.068
30	0.6	0.7	0.588	0.587	0.089	0.090	0.857	0.208	0.128	0.128	0.856	0.076
30	0.8	0.5	0.792	0.793	0.077	0.077	0.474	0.034	0.078	0.08	0.479	0.024
30	0.8	0.7	0.783	0.785	0.076	0.075	0.575	0.168	0.108	0.108	0.570	0.026
100	0.0	0.0	0.001	0.002	0.056	0.056	0.005	0.044	0.065	0.057	0.172	0.046
100	0.0	0.1	-0.004	-0.004	0.055	0.055	-0.086	0.044	0.059	0.060	0.119	0.054
100	0.0	0.3	-0.002	0.000	0.055	0.055	-0.036	0.080	0.069	0.065	0.063	0.048
100	0.2	0.1	0.197	0.193	0.058	0.058	0.106	0.036	0.056	0.055	0.154	0.050
100	0.2	0.3	0.196	0.196	0.058	0.058	0.126	0.036	0.06	0.057	0.272	0.042
100	0.2	0.5	0.194	0.199	0.058	0.058	0.294	0.088	0.071	0.071	0.409	0.052
100	0.2	0.7	0.201	0.198	0.058	0.058	0.074	0.136	0.073	0.075	0.110	0.038
100	0.4	0.1	0.396	0.400	0.056	0.056	0.348	0.090	0.066	0.067	0.395	0.052
100	0.4	0.3	0.394	0.398	0.056	0.056	0.381	0.044	0.064	0.061	0.473	0.038
100	0.4	0.5	0.399	0.401	0.056	0.056	0.394	0.082	0.063	0.055	0.375	0.066
100	0.4	0.7	0.393	0.388	0.056	0.056	0.403	0.104	0.071	0.071	0.513	0.070
100	0.6	0.3	0.596	0.596	0.051	0.051	0.395	0.052	0.054	0.053	0.409	0.040
100	0.6	0.5	0.599	0.603	0.050	0.050	0.531	0.070	0.062	0.062	0.645	0.058
100	0.6	0.7	0.594	0.596	0.050	0.050	0.839	0.136	0.071	0.071	0.838	0.046
100	0.8	0.5	0.798	0.801	0.042	0.042	0.486	0.052	0.043	0.044	0.496	0.040
100	0.8	0.7	0.800	0.798	0.042	0.042	0.568	0.138	0.059	0.059	0.564	0.042

Table 10: Bootstrap method. Simulation results obtained for a 3-ordinal scale with uniform marginal distributions when $n_k = 2$

Parameters					Delta method - classic				Bootstrap - multilevel			
K	κ	INT	κ_1	κ_2	$SE(\kappa_1)$	$SE(\kappa_2)$	$CORR$	α	$SE(\kappa_1)$	$SE(\kappa_2)$	$CORR$	α
20	0.0	0.0	-0.003	-0.005	0.120	0.120	-0.008	0.048	0.137	0.121	0.134	0.056
20	0.0	0.1	-0.003	-0.009	0.120	0.118	-0.087	0.056	0.125	0.125	0.087	0.054
20	0.0	0.3	-0.002	-0.019	0.118	0.117	-0.058	0.100	0.141	0.133	0.051	0.076
20	0.2	0.1	0.191	0.198	0.127	0.127	0.107	0.038	0.120	0.117	0.160	0.046
20	0.2	0.3	0.196	0.195	0.126	0.126	0.143	0.032	0.127	0.123	0.285	0.046
20	0.2	0.5	0.200	0.204	0.123	0.123	0.276	0.110	0.148	0.147	0.380	0.078
20	0.2	0.7	0.201	0.174	0.122	0.123	0.074	0.130	0.153	0.157	0.125	0.048
20	0.4	0.1	0.393	0.380	0.123	0.122	0.354	0.098	0.142	0.145	0.402	0.064
20	0.4	0.3	0.398	0.393	0.122	0.122	0.372	0.072	0.136	0.131	0.458	0.074
20	0.4	0.5	0.398	0.393	0.122	0.123	0.397	0.056	0.133	0.120	0.395	0.044
20	0.4	0.7	0.393	0.384	0.119	0.121	0.391	0.108	0.149	0.149	0.507	0.060
20	0.6	0.3	0.586	0.586	0.111	0.111	0.383	0.060	0.118	0.117	0.401	0.046
20	0.6	0.5	0.593	0.594	0.109	0.109	0.521	0.070	0.132	0.133	0.648	0.050
20	0.6	0.7	0.588	0.589	0.107	0.107	0.875	0.236	0.152	0.151	0.873	0.060
20	0.8	0.5	0.800	0.800	0.082	0.082	0.441	0.032	0.092	0.094	0.517	0.020
20	0.8	0.7	0.768	0.778	0.086	0.084	0.521	0.216	0.124	0.122	0.526	0.028
30	0.0	0.0	-0.001	-0.006	0.099	0.099	-0.018	0.070	0.113	0.100	0.128	0.074
30	0.0	0.1	0.000	-0.004	0.099	0.099	-0.082	0.076	0.104	0.105	0.117	0.090
30	0.0	0.3	-0.013	-0.012	0.098	0.098	-0.047	0.086	0.119	0.111	0.060	0.058
30	0.2	0.1	0.189	0.190	0.104	0.104	0.109	0.032	0.100	0.097	0.171	0.052
30	0.2	0.3	0.188	0.201	0.104	0.103	0.131	0.05	0.106	0.100	0.287	0.070
30	0.2	0.5	0.185	0.189	0.102	0.102	0.285	0.092	0.124	0.123	0.399	0.064
30	0.2	0.7	0.187	0.190	0.102	0.102	0.071	0.116	0.128	0.131	0.11	0.062
30	0.4	0.1	0.405	0.398	0.101	0.101	0.338	0.104	0.117	0.120	0.375	0.054
30	0.4	0.3	0.392	0.388	0.101	0.101	0.377	0.044	0.113	0.109	0.469	0.050
30	0.4	0.5	0.387	0.389	0.101	0.101	0.388	0.092	0.110	0.099	0.366	0.068
30	0.4	0.7	0.393	0.396	0.100	0.100	0.407	0.100	0.126	0.124	0.52	0.052
30	0.6	0.3	0.587	0.593	0.092	0.091	0.390	0.058	0.099	0.095	0.412	0.052
30	0.6	0.5	0.594	0.595	0.090	0.090	0.519	0.064	0.109	0.111	0.644	0.066
30	0.6	0.7	0.588	0.587	0.089	0.090	0.857	0.208	0.126	0.127	0.858	0.076
30	0.8	0.5	0.792	0.793	0.077	0.077	0.474	0.034	0.079	0.081	0.476	0.026
30	0.8	0.7	0.783	0.785	0.076	0.075	0.575	0.168	0.109	0.108	0.575	0.028
100	0.0	0.0	0.001	0.002	0.056	0.056	0.005	0.044	0.064	0.057	0.170	0.040
100	0.0	0.1	-0.004	-0.004	0.055	0.055	-0.086	0.044	0.058	0.060	0.116	0.056
100	0.0	0.3	-0.002	0.000	0.055	0.055	-0.036	0.080	0.068	0.064	0.064	0.052
100	0.2	0.1	0.197	0.193	0.058	0.058	0.106	0.036	0.056	0.054	0.156	0.046
100	0.2	0.3	0.196	0.196	0.058	0.058	0.126	0.036	0.059	0.057	0.271	0.044
100	0.2	0.5	0.194	0.199	0.058	0.058	0.294	0.088	0.070	0.071	0.407	0.052
100	0.2	0.7	0.201	0.198	0.058	0.058	0.074	0.136	0.073	0.075	0.112	0.042
100	0.4	0.1	0.396	0.400	0.056	0.056	0.348	0.090	0.066	0.067	0.394	0.052
100	0.4	0.3	0.394	0.398	0.056	0.056	0.381	0.044	0.063	0.061	0.472	0.0380
100	0.4	0.5	0.399	0.401	0.056	0.056	0.394	0.082	0.062	0.055	0.377	0.066
100	0.4	0.7	0.393	0.388	0.056	0.056	0.403	0.104	0.071	0.070	0.513	0.070
100	0.6	0.3	0.596	0.596	0.051	0.051	0.395	0.052	0.054	0.053	0.409	0.042
100	0.6	0.5	0.599	0.603	0.050	0.050	0.531	0.070	0.061	0.062	0.645	0.058
100	0.6	0.7	0.594	0.596	0.050	0.050	0.839	0.136	0.071	0.071	0.840	0.046
100	0.8	0.5	0.798	0.801	0.042	0.042	0.486	0.052	0.043	0.044	0.495	0.042
100	0.8	0.7	0.800	0.798	0.042	0.042	0.568	0.138	0.059	0.059	0.565	0.040

Table 11: Delta method. Simulation results obtained for a 3-ordinal scale with uniform marginal distributions when $n_k = 3$

Parameters					Delta method - classic				Delta - multilevel			
K	κ	INT	κ_1	κ_2	$SE(\kappa_1)$	$SE(\kappa_2)$	$CORR$	α	$SE(\kappa_1)$	$SE(\kappa_2)$	$CORR$	α
20	0.0	0.0	-0.010	-0.019	0.100	0.099	0.007	0.024	0.107	0.103	0.279	0.054
20	0.0	0.1	-0.010	-0.013	0.099	0.099	-0.066	0.052	0.103	0.117	0.152	0.054
20	0.0	0.3	0.001	-0.013	0.097	0.097	0.143	0.042	0.136	0.124	0.558	0.040
20	0.2	0.1	0.182	0.182	0.104	0.104	0.208	0.054	0.120	0.116	0.382	0.054
20	0.2	0.3	0.190	0.186	0.103	0.102	0.206	0.058	0.113	0.133	0.451	0.058
20	0.2	0.5	0.191	0.197	0.102	0.102	0.296	0.136	0.141	0.142	0.443	0.054
20	0.2	0.7	0.184	0.200	0.100	0.100	0.238	0.216	0.161	0.157	0.350	0.082
20	0.4	0.1	0.386	0.391	0.102	0.102	0.271	0.060	0.108	0.114	0.339	0.058
20	0.4	0.3	0.377	0.384	0.101	0.101	0.349	0.044	0.123	0.118	0.572	0.052
20	0.4	0.5	0.388	0.383	0.100	0.100	0.360	0.064	0.130	0.130	0.640	0.046
20	0.4	0.7	0.393	0.387	0.098	0.100	0.326	0.222	0.142	0.143	0.285	0.052
20	0.6	0.3	0.587	0.595	0.092	0.091	0.466	0.056	0.092	0.102	0.493	0.056
20	0.6	0.5	0.584	0.588	0.090	0.090	0.489	0.150	0.131	0.127	0.585	0.052
20	0.6	0.7	0.589	0.593	0.088	0.087	0.444	0.306	0.155	0.153	0.437	0.038
20	0.8	0.5	0.795	0.798	0.069	0.069	0.461	0.046	0.078	0.075	0.559	0.030
20	0.8	0.7	0.783	0.788	0.068	0.067	0.926	0.238	0.120	0.118	0.925	0.028
30	0.0	0.0	-0.013	-0.006	0.082	0.082	-0.008	0.038	0.086	0.085	0.268	0.052
30	0.0	0.1	-0.009	-0.013	0.082	0.082	-0.066	0.044	0.085	0.096	0.142	0.050
30	0.0	0.3	-0.010	-0.007	0.081	0.081	0.162	0.042	0.113	0.103	0.582	0.064
30	0.2	0.1	0.193	0.191	0.086	0.086	0.202	0.076	0.100	0.096	0.36	0.058
30	0.2	0.3	0.197	0.194	0.085	0.085	0.220	0.062	0.094	0.111	0.470	0.062
30	0.2	0.5	0.189	0.195	0.084	0.084	0.299	0.118	0.117	0.117	0.448	0.058
30	0.2	0.7	0.199	0.196	0.083	0.083	0.249	0.220	0.135	0.131	0.355	0.062
30	0.4	0.1	0.398	0.396	0.084	0.084	0.264	0.058	0.090	0.093	0.338	0.054
30	0.4	0.3	0.394	0.395	0.083	0.083	0.355	0.054	0.102	0.097	0.585	0.064
30	0.4	0.5	0.394	0.391	0.082	0.082	0.388	0.034	0.107	0.107	0.663	0.042
30	0.4	0.7	0.395	0.394	0.082	0.082	0.316	0.190	0.119	0.117	0.276	0.056
30	0.6	0.3	0.594	0.592	0.075	0.075	0.461	0.078	0.074	0.084	0.495	0.074
30	0.6	0.5	0.593	0.591	0.074	0.074	0.473	0.106	0.106	0.104	0.561	0.04
30	0.6	0.7	0.584	0.587	0.073	0.073	0.438	0.290	0.128	0.127	0.432	0.06
30	0.8	0.5	0.795	0.794	0.057	0.057	0.474	0.058	0.065	0.063	0.56	0.054
30	0.8	0.7	0.792	0.795	0.056	0.055	0.897	0.268	0.098	0.096	0.897	0.036
100	0.0	0.0	-0.004	0.001	0.045	0.045	0.005	0.024	0.048	0.047	0.289	0.052
100	0.0	0.1	-0.005	-0.006	0.045	0.045	-0.058	0.060	0.047	0.054	0.166	0.064
100	0.0	0.3	-0.005	-0.002	0.045	0.045	0.193	0.034	0.063	0.058	0.612	0.056
100	0.2	0.1	0.198	0.199	0.047	0.047	0.219	0.050	0.055	0.053	0.396	0.044
100	0.2	0.3	0.202	0.197	0.047	0.047	0.235	0.048	0.052	0.062	0.489	0.050
100	0.2	0.5	0.193	0.201	0.047	0.047	0.308	0.126	0.065	0.065	0.447	0.066
100	0.2	0.7	0.193	0.194	0.047	0.047	0.242	0.206	0.075	0.073	0.339	0.064
100	0.4	0.1	0.400	0.396	0.046	0.046	0.267	0.054	0.049	0.051	0.324	0.046
100	0.4	0.3	0.399	0.401	0.046	0.046	0.358	0.052	0.057	0.054	0.585	0.054
100	0.4	0.5	0.398	0.397	0.046	0.046	0.381	0.052	0.059	0.060	0.659	0.058
100	0.4	0.7	0.401	0.393	0.046	0.046	0.306	0.192	0.066	0.066	0.264	0.044
100	0.6	0.3	0.597	0.596	0.041	0.041	0.463	0.056	0.041	0.046	0.504	0.054
100	0.6	0.5	0.600	0.600	0.041	0.041	0.477	0.122	0.059	0.058	0.565	0.042
100	0.6	0.7	0.595	0.595	0.041	0.041	0.399	0.262	0.071	0.071	0.394	0.070
100	0.8	0.5	0.797	0.797	0.032	0.031	0.470	0.054	0.036	0.034	0.561	0.054
100	0.8	0.7	0.795	0.795	0.031	0.031	0.850	0.276	0.054	0.054	0.849	0.054

Table 12: Bootstrap method. Simulation results obtained for a 3-ordinal scale with uniform marginal distributions when $n_k = 3$

Parameters					Delta method - classic				Bootstrap - multilevel			
K	κ	INT	κ_1	κ_2	$SE(\kappa_1)$	$SE(\kappa_2)$	$CORR$	α	$SE(\kappa_1)$	$SE(\kappa_2)$	$CORR$	α
20	0.0	0.0	-0.010	-0.019	0.100	0.099	0.007	0.024	0.104	0.100	0.268	0.058
20	0.0	0.1	-0.010	-0.013	0.099	0.099	-0.066	0.052	0.101	0.114	0.146	0.060
20	0.0	0.3	0.001	-0.013	0.097	0.097	0.143	0.042	0.131	0.121	0.546	0.036
20	0.2	0.1	0.182	0.182	0.104	0.104	0.208	0.054	0.118	0.113	0.376	0.064
20	0.2	0.3	0.190	0.186	0.103	0.102	0.206	0.058	0.111	0.129	0.444	0.058
20	0.2	0.5	0.191	0.197	0.102	0.102	0.296	0.136	0.137	0.137	0.440	0.056
20	0.2	0.7	0.184	0.200	0.100	0.100	0.238	0.216	0.156	0.152	0.354	0.088
20	0.4	0.1	0.386	0.391	0.102	0.102	0.271	0.060	0.107	0.112	0.339	0.068
20	0.4	0.3	0.377	0.384	0.101	0.101	0.349	0.044	0.120	0.115	0.562	0.052
20	0.4	0.5	0.388	0.383	0.100	0.100	0.360	0.064	0.126	0.126	0.632	0.046
20	0.4	0.7	0.393	0.387	0.098	0.100	0.326	0.222	0.139	0.139	0.293	0.062
20	0.6	0.3	0.587	0.595	0.092	0.091	0.466	0.056	0.091	0.101	0.494	0.062
20	0.6	0.5	0.584	0.588	0.090	0.090	0.489	0.150	0.128	0.124	0.586	0.058
20	0.6	0.7	0.589	0.593	0.088	0.087	0.444	0.306	0.152	0.150	0.447	0.044
20	0.8	0.5	0.795	0.798	0.069	0.069	0.461	0.046	0.078	0.075	0.554	0.044
20	0.8	0.7	0.783	0.788	0.068	0.067	0.926	0.238	0.119	0.117	0.926	0.032
30	0.0	0.0	-0.013	-0.006	0.082	0.082	-0.008	0.038	0.084	0.084	0.262	0.056
30	0.0	0.1	-0.009	-0.013	0.082	0.082	-0.066	0.044	0.084	0.094	0.139	0.056
30	0.0	0.3	-0.010	-0.007	0.081	0.081	0.162	0.042	0.110	0.101	0.574	0.056
30	0.2	0.1	0.193	0.191	0.086	0.086	0.202	0.076	0.098	0.094	0.354	0.062
30	0.2	0.3	0.197	0.194	0.085	0.085	0.220	0.062	0.093	0.109	0.465	0.062
30	0.2	0.5	0.189	0.195	0.084	0.084	0.299	0.118	0.114	0.115	0.447	0.054
30	0.2	0.7	0.199	0.196	0.083	0.083	0.249	0.220	0.131	0.128	0.358	0.064
30	0.4	0.1	0.398	0.396	0.084	0.084	0.264	0.058	0.089	0.092	0.337	0.054
30	0.4	0.3	0.394	0.395	0.083	0.083	0.355	0.054	0.101	0.096	0.578	0.066
30	0.4	0.5	0.394	0.391	0.082	0.082	0.388	0.034	0.105	0.106	0.658	0.042
30	0.4	0.7	0.395	0.394	0.082	0.082	0.316	0.190	0.116	0.115	0.280	0.06
30	0.6	0.3	0.594	0.592	0.075	0.075	0.461	0.078	0.074	0.084	0.496	0.078
30	0.6	0.5	0.593	0.591	0.074	0.074	0.473	0.106	0.105	0.102	0.563	0.042
30	0.6	0.7	0.584	0.587	0.073	0.073	0.438	0.290	0.126	0.125	0.439	0.068
30	0.8	0.5	0.795	0.794	0.057	0.057	0.474	0.058	0.065	0.063	0.557	0.056
30	0.8	0.7	0.792	0.795	0.056	0.055	0.897	0.268	0.097	0.096	0.90	0.040
100	0.0	0.0	-0.004	0.001	0.045	0.045	0.005	0.024	0.048	0.047	0.285	0.056
100	0.0	0.1	-0.005	-0.006	0.045	0.045	-0.058	0.06	0.047	0.053	0.163	0.064
100	0.0	0.3	-0.005	-0.002	0.045	0.045	0.193	0.034	0.063	0.057	0.61	0.056
100	0.2	0.1	0.198	0.199	0.047	0.047	0.219	0.050	0.055	0.053	0.394	0.044
100	0.2	0.3	0.202	0.197	0.047	0.047	0.235	0.048	0.052	0.061	0.488	0.050
100	0.2	0.5	0.193	0.201	0.047	0.047	0.308	0.126	0.065	0.065	0.446	0.068
100	0.2	0.7	0.193	0.194	0.047	0.047	0.242	0.206	0.075	0.073	0.34	0.066
100	0.4	0.1	0.400	0.396	0.046	0.046	0.267	0.054	0.049	0.051	0.324	0.044
100	0.4	0.3	0.399	0.401	0.046	0.046	0.358	0.052	0.057	0.054	0.583	0.056
100	0.4	0.5	0.398	0.397	0.046	0.046	0.381	0.052	0.059	0.059	0.656	0.060
100	0.4	0.7	0.401	0.393	0.046	0.046	0.306	0.192	0.066	0.065	0.266	0.050
100	0.6	0.3	0.597	0.596	0.041	0.041	0.463	0.056	0.041	0.046	0.504	0.054
100	0.6	0.5	0.600	0.600	0.041	0.041	0.477	0.122	0.059	0.058	0.566	0.044
100	0.6	0.7	0.595	0.595	0.041	0.041	0.399	0.262	0.071	0.071	0.396	0.072
100	0.8	0.5	0.797	0.797	0.032	0.031	0.470	0.054	0.036	0.034	0.560	0.052
100	0.8	0.7	0.795	0.795	0.031	0.031	0.850	0.276	0.054	0.054	0.851	0.056

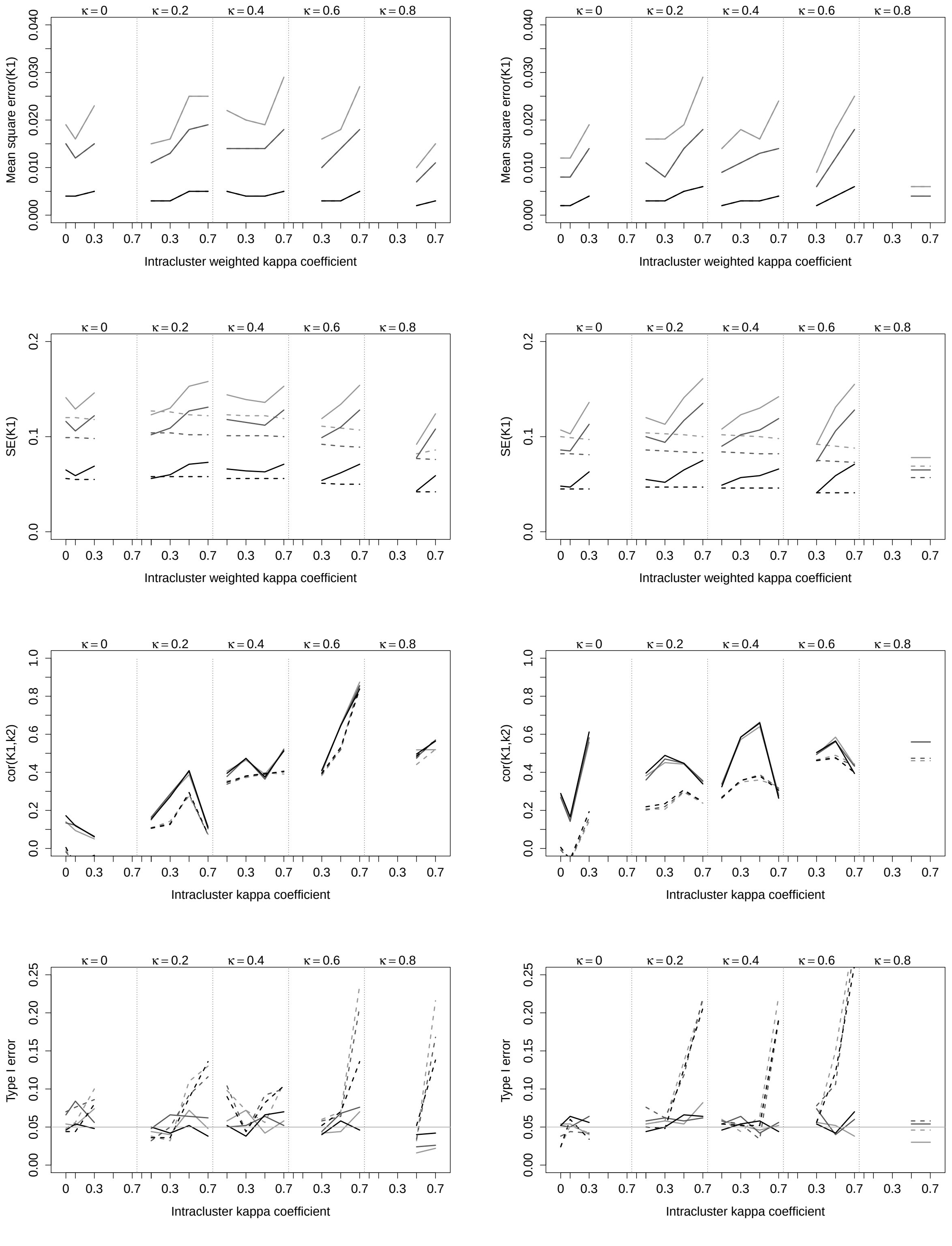

Figure 3: Mean squared error and mean standard error of κ_1, mean correlation between κ_1 and κ_2 and type I error for the comparison of the two dependent multilevel kappa coefficients obtained on a 3-ordinal scale when the observers marginal probability distribution is uniform and the cluster size is equal to $n_k = 2$ (left) and $n_k = 3$ (right). The results obtained by the delta method ignoring the hierarchical structure (dashed lines) and by the hierarchical delta method (plain line) are reported for $K = 100$ (black), $K = 30$ (middle gray) and $K = 20$ (light gray) clusters
