# Supplementary material for: Comparing dependent kappa coefficients obtained on multilevel data
Source: Biom J. 2017 May 2;59(5):1016–34. doi: 10.1002/bimj.201600093 (PMC5600130; doi:10.1002/bimj.201600093)
Supplement: Supplementary file 2 — Supporting Information. [file BIMJ-59-1016-s002.zip › CODE_AND_DATA/Example/FEES_data.pdf]

Description of the dataset FEES_wide

More information on the context of the study can be found in

Pilz,W., S. Vanbelle, B. Kremer, M. van Hooren, T. van Becelaere, N. Roodenburg, and L. Baijens (2016). Observers' agreement on measurements in fiberoptic endoscopic evaluation of swallowing. *Dysphagia* 31(2), 180–187.

Column 1. subject: identification number of the subject from 1 to 37

Column 2. group: dysphagia ethiology (1 = head and neck oncological, 2 = neurological)

Column 3. swallow: liquid consistency (1 = thin, 4 = thick)

Column 4. piecemeal: first assessment of piecemeal deglutition (0 = normal, 1 = two swallows, 2 = three swallows, 3 = four swallows, 4 = five or more swallows).

Column 5. piecemealRM: second assessment of piecemeal deglutition (0 = normal, 1 = two swallows, 2 = three swallows, 3 = four swallows, 4 = five or more swallows).

Column 6. valleculae: first assessment of valleculae pooling (0 = normal, 1 = <50%, 2 = >50%).

Column 7. valleculaeRM: second assessment of valleculae pooling (0 = normal, 1 = <50%, 2 = >50%).

Column 8. pyriform: first assessment of pyriform pooling (0 = normal, 1 = moderate, 2 = severe).

Column 9. pyriformRM: second assessment of pyriform pooling (0 = normal, 1 = moderate, 2 = severe).

Column 10. penasp: first assessment of penetration/aspiration (0 = normal, 1 = penetration, 2 = aspiration).

Column 11. pyriformRM: second assessment of penetration/aspiration (0 = normal, 1 = penetration, 2 = aspiration).

Column 12. R1: binary variable to identify rater 1 (1 = rater 1, 0 = other rater).

Column 13. R2: binary variable to identify rater 2 (1 = rater 2, 0 = other rater).

Column 14. CON: binary variable to identify consensus rating (1 = consensus, 0 = other rater).
